# Downy mildew resistance induced by *Trichoderma harzianum* T39 in susceptible grapevines partially mimics transcriptional changes of resistant genotypes

**DOI:** 10.1186/1471-2164-13-660

**Published:** 2012-11-22

**Authors:** Michele Perazzolli, Marco Moretto, Paolo Fontana, Alberto Ferrarini, Riccardo Velasco, Claudio Moser, Massimo Delledonne, Ilaria Pertot

**Affiliations:** 1 IASMA Research and Innovation Centre, Fondazione Edmund Mach, Via E. Mach 1, 38010, San Michele all’Adige (TN), Italy; 2 Dipartimento di Biotecnologie, Università degli Studi di Verona, Strada Le Grazie 15, 37134, Verona, Italy

**Keywords:** Induced resistance, Next generation sequencing, RNA-Seq, Transcriptomics, Gene expression, *Vitis vinifera*, Plant-pathogen interactions

## Abstract

**Background:**

Downy mildew, caused by *Plasmopara viticola*, is one of the most severe diseases of grapevine and is commonly controlled by fungicide treatments. The beneficial microorganism *Trichoderma harzianum* T39 (T39) can induce resistance to downy mildew, although the molecular events associated with this process have not yet been elucidated in grapevine. A next generation RNA sequencing (RNA-Seq) approach was used to study global transcriptional changes associated with resistance induced by T39 in *Vitis vinifera* Pinot Noir leaves. The long-term aim was to develop strategies to optimize the use of this agent for downy mildew control.

**Results:**

More than 14.8 million paired-end reads were obtained for each biological replicate of T39-treated and control leaf samples collected before and 24 h after *P*. *viticola* inoculation. RNA-Seq analysis resulted in the identification of 7,024 differentially expressed genes, highlighting the complex transcriptional reprogramming of grapevine leaves during resistance induction and in response to pathogen inoculation. Our data show that T39 has a dual effect: it directly modulates genes related to the microbial recognition machinery, and it enhances the expression of defence-related processes after pathogen inoculation. Whereas several genes were commonly affected by *P*. *viticola* in control and T39-treated plants, opposing modulation of genes related to responses to stress and protein metabolism was found. T39-induced resistance partially inhibited some disease-related processes and specifically activated defence responses after *P*. *viticola* inoculation, causing a significant reduction of downy mildew symptoms.

**Conclusions:**

The global transcriptional analysis revealed that defence processes known to be implicated in the reaction of resistant genotypes to downy mildew were partially activated by T39-induced resistance in susceptible grapevines. Genes identified in this work are an important source of markers for selecting novel resistance inducers and for the analysis of environmental conditions that might affect induced resistance mechanisms.

## Background

*Plasmopara viticola* (Berk. and Curt.) Berl. and de Toni is a biotrophic oomycete that causes downy mildew in grapevine [1]. This devastating disease occurs worldwide, particularly in regions with warm and wet conditions during the growing season. *P*. *viticola* mainly infects leaves and clusters of young berries and produces oil spot lesions on the adaxial leaf surface accompanied by massive sporulation on the abaxial surface. Although downy mildew can be controlled by frequent applications of chemical fungicides, concerns about the environmental impact of pesticide overuse [2] and the development of resistant *P*. *viticola* populations [3] have sparked an interest in alternatives to chemical treatments.

The grapevine industry relies predominantly on *Vitis vinifera*, which is susceptible to downy mildew. Resistance traits have been identified in wild grapevine species, and the mechanisms of resistance to downy mildew have been characterized in resistant genotypes [1]. Transcriptomic analysis supports the view that downy mildew resistance is mainly a post-infection phenomenon [4] and highlights the importance of transcriptional reprogramming in both resistant and susceptible genotypes in response to *P*. *viticola* inoculation [4-8]. Transcriptional changes associated with *P*. *viticola* infection of susceptible grapevines have been related to a weak defence response [4] and to the establishment of a compatible interaction [5,7,9,10]. The response of resistant genotypes has been characterized by strong and rapid transcriptional reprogramming of processes related to defence, signal transduction, and secondary metabolism, which are either not induced or induced to a lesser extent in susceptible grapevines [4,8,11-14]. In particular, downy mildew resistance has been correlated with enhanced expression of genes encoding pathogenesis-related (PR) proteins and enzymes of phenylpropanoid biosynthesis, and with specific modulation of signal transduction components and markers of hypersensitive response (HR) in resistant grapevines [4,8,11-13].

Downy mildew symptoms can be significantly reduced in susceptible grapevines by preventive application of resistance inducers, such as chitosan [15], laminarins [16-18], β-aminobutyric acid (BABA) [19,20], acibenzolar-S-methyl (BTH) [21,22], and thiamine [23]. Treatments with plant extracts [24] or microbial extracts [22,25] have also been found to increase grapevine resistance to downy mildew. The ascomycete *Trichoderma harzianum* strain T39 (T39) significantly reduces downy mildew symptoms by activating grapevine resistance both locally and systemically [21], although the molecular events responsible for resistance induction have not yet been clarified. *Trichoderma* spp. strains have been characterized in model systems based on their ability to induce plant resistance against pathogens [26,27] by reprogramming the plant transcriptome [28-32]. Specific strains of beneficial microorganisms can improve plant performance by activating a plant-mediated defence mechanism known as induced systemic resistance (ISR) [33]. Through root or leaf interactions [21,34], beneficial microorganisms are recognized by the plant, which results in a mild but effective activation of the plant immune responses in all tissues [35]. ISR confers broad-spectrum resistance to various types of pathogens and abiotic stresses [27,36] and is usually regulated by jasmonic acid (JA)- and ethylene (ET)-dependent signalling pathways [33]. Rather than directly activating plant defences, beneficial microorganisms that induce resistance usually prime the plant so that it responds more strongly upon exposure to the stress condition [35,36]. Primed plants display faster and/or stronger activation of the defence responses after pathogen inoculation [37]. Because plant defences are activated only when they are really needed [38], priming provides advantages in terms of energy costs for the plant [39], and it is probably evolved to save energy under pathogen-free conditions [40]. The benefits of priming outweigh its costs when disease does occur, and priming is seen as a promising strategy in modern disease management [41]. The absence of apparent energy costs associated with T39-induced resistance in grapevine suggested a priming state activation [42]. However, the molecular mechanisms underlying resistance induction have been only partially revealed [26,43], and information regarding induced resistance in grapevine is particularly scarce [20,44].

In this study, we used Illumina RNA-Seq analysis to characterize the global transcriptional dynamics associated with T39-induced resistance in grapevine. To the best of our knowledge, this study is the first to use high-throughput sequencing technology to investigate molecular events underlying induced resistance in plants, and it is also the first transcriptome-wide characterization of resistance induced by a beneficial microbe in a non-model plant. Our analysis revealed that the reduction of downy mildew symptoms is related to a complex transcriptional reprogramming in T39-treated plants, both before and 24 h after pathogen inoculation. In particular, the reaction of T39-treated plants to pathogen inoculation is associated with enhanced expression of *P*. *viticola*-responsive genes and specific modulation of some genes related to defence in resistant grapevines. Our study has identified genes that could be valuable as markers of ISR activation for subsequent selection of new resistance inducers with improved ability to stimulate plant defences.

## Results

### RNA-Seq sequencing and mapping of reads to the grapevine genome

**Figure 1:**
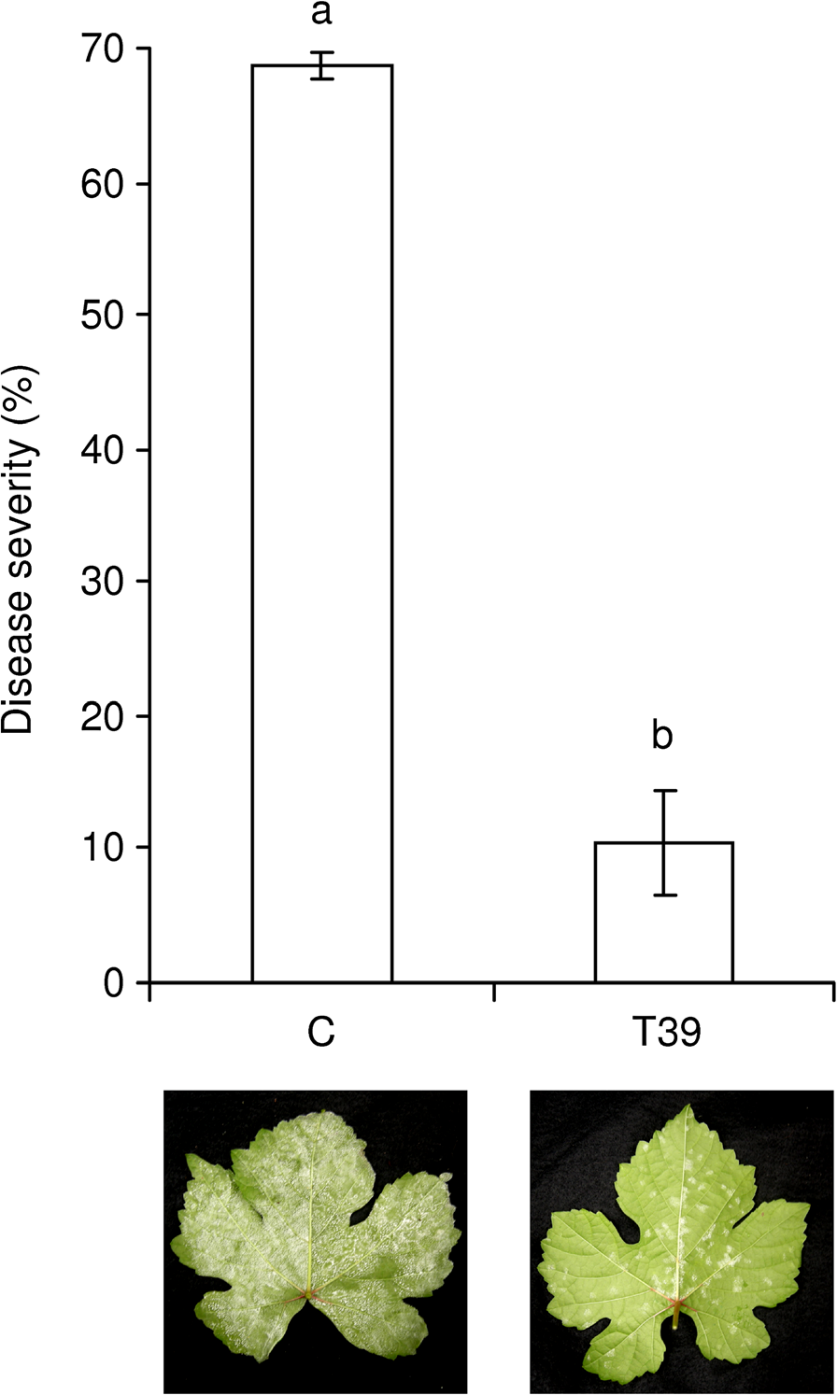
**Grapevine resistance induced by treatment with *Trichoderma harzianum *T39. **Downy mildew severity (%) was assessed on *Vitis vinifera* Pinot Noir control plants (C) and plants treated with *T*. *harzianum* T39 (T39). Mean severity scores and standard errors for six replicates are presented. Treatments followed by different letters are significantly different according to Tukey’s test (*P* < 0.05). Photographs show sporulation of downy mildew on representative leaves.

The ability of T39 to activate local and systemic resistance processes has been previously reported in grapevine [21,42], and leaf treatment was used to study responses to T39 treatment and pathogen inoculation in the same plant organ. Leaf samples were collected before and 24 h after *P*. *viticola* inoculation from T39-treated and control plants, and resistance induction was confirmed by phenotypic analysis (Figure 1). Four different treatments were analysed by RNA-Seq: control (C), T39-treated (T39), *P*. *viticola*-inoculated control (C+*P*.*v*.) and *P*. *viticola*-inoculated T39-treated (T39+*P*.*v*.) plants. Three biological replicates of each treatment were analysed, with each biological replicate sequenced twice in separate lanes (sequencing replicate). Between 6.1 and 18.8 million paired-end reads of 100 nucleotides were obtained for each sequencing replicate, and an average of 94% of these passed the quality control test (filtered reads; Additional files 1 and 2). Summing the reads of two sequencing replicates, more than 14.8 million filtered reads were obtained for each biological replicate (Table 1), corresponding to a coverage of at least 32× the grapevine transcriptome (Table 2).

**Table 1:** RNA-Seq sequencing and read mapping to the grapevine genome

**Treatment**^**a**^	**Replicate**^**b**^	**Total reads**^**c**^	**Filtered reads**^**d**^	**%**	**Mapped reads**^**e**^	**%**	**Unique reads**^**f**^	**%**	**Multi**-**reads**^**g**^	**%**
C	1	20480361	19388264	95	17975427	93	17473619	97	501808	3
	2	20182309	19095859	95	17935380	94	17392482	97	542898	3
	3	15856292	14845528	94	13327853	90	12749078	96	578775	4
T39	1	24312739	23208015	95	21103884	91	20207496	96	896388	4
	2	35315408	32565826	92	30409086	93	29495812	97	913274	3
	3	25081653	23722385	95	21873968	92	21145558	97	728410	3
C+*P*.*v*.	1	21934228	20800485	95	19240139	92	18603657	97	636482	3
	2	25097152	23684317	94	21637677	91	20896985	97	740692	3
	3	22471334	21555054	96	19384445	90	18634940	96	749505	4
T39+*P*.*v*.	1	22897386	21318685	93	18406407	86	17576560	95	829847	5
	2	29049603	26710912	92	24801196	93	23981057	97	820139	3
	3	22603588	21369934	95	19558774	92	18859902	96	698872	4

^a^ Grapevine leaves of control (C), *Trichoderma harzianum* T39-treated (T39), *Plasmopara viticola*-inoculated control (C+*P*.*v*.) and *P*. *viticola*-inoculated T39-treated (T39+*P*.*v.*) plants.

^b^ Biological replicates (plants), numbered from 1 to 3.

^c^ Total sequenced reads as sum of reads obtained from two replicates of sequencing for each treatment.

^d^ Reads passing the quality check and the corresponding percentage (%) of total reads.

^e^ Reads mapping to Pinot Noir grapevine genome [77] Release 3 [78], and the corresponding percentage (%) of filtered reads.

^f^ Reads mapping to unique locations in the grapevine genome, and the corresponding percentage (%) of mapped reads.

^g^ Reads mapping to more than one location (2–100 matches) in the grapevine genome, and the corresponding percentage (%) of mapped reads.

**Table 2:** **RNA**-**Seq sequencing and coverage of the grapevine transcriptome for each biological replicate**

**Treatment**^**a**^	**Replicate**^**b**^	**Sequenced bases(Mbp)**^**c**^	**Read length(bp)**^**d**^	**Coverage (fold)**^**e**^
C	1	1844	95	42
	2	1812	95	42
	3	1393	93	32
T39	1	2195	94	50
	2	3054	94	70
	3	2253	95	52
C+*P*.*v*.	1	1981	95	46
	2	2244	95	52
	3	2049	95	47
T39+*P*.*v*.	1	1999	94	46
	2	2500	94	57
	3	2028	95	47

^a^ Grapevine leaves of control (C), *Trichoderma harzianum* T39-treated (T39), *Plasmopara viticola*-inoculated control (C+*P*.*v*.) and *P*. *viticola*-inoculated T39-treated (T39+*P*.*v.*) plants.

^b^ Biological replicates (plants), numbered from 1 to 3.

^c^ Total bases (Mbp) sequenced by RNA-Seq analysis that passed the quality check.

^d^ Mean length (bp) of sequenced reads.

^e^ Coverage of the Pinot Noir grapevine transcriptome [77] Release 3 (43.5 Mbp) [78].

An average of 91% of filtered reads mapped to the grapevine genome (mapped reads) for each biological replicate (Table 1), and similar percentages mapped for each sequencing replicate (Additional file 1). An average of 96% of mapped reads matched to unique locations (unique reads), 4% displayed multiple matches (multi-reads) to the grapevine genome (Table 1 and Additional files 1 and 3), and about 77% of mapped reads matched to grapevine genes (Additional files 3 and 4).

### Gene expression estimation by RNA-Seq

Gene expression level was assessed on the basis of both unique and multi-reads (Additional file 3) to improve evaluation of members of multigene families [45,46], and it was expressed as fragments per kilobase of transcript per million fragments mapped (FPKM) values using Cufflinks [46]. Between one and nine transcript isoforms were recognized for each grapevine gene, and 3,679 to 7,548 novel genes were identified in each sequencing replicate (Additional file 5), representing important new information for genome annotation. About 66% of grapevine genes were expressed in each sequencing replicate (Additional files 5 and 6). High correlations between sequencing replicates were obtained (Pearson’s correlation coefficients and R^2^ values greater than 0.98 and 0.95, respectively; Additional files 7, 8 and 9), and read counts were summed to obtain better coverage and improve variance estimation of each biological replicate [47]. Considering the expression of all grapevine genes, the Pearson’s correlation coefficient between control and T39-treated plants (T39 *vs*. C) was greater than that between control and *P*. *viticola*-inoculated plants (C+*P*.*v*. *vs*. C and T39+*P*.*v*. *vs*. C; Table 3), suggesting that few transcriptional changes were caused by T39 treatment and that major transcriptional reprogramming occurred after pathogen inoculation.

**Table 3:** **Pearson**’**s correlation coefficients between grapevine treatments**

**Comparison**	**Pearson’s correlation coefficient**
C vs. T39	0.93
C vs. C+*P*.*v*.	0.83
C vs. T39+*P*.*v*.	0.80
T39 vs. C+*P*.*v*.	0.80
T39 vs. T39+*P*.*v*.	0.81
C+*P*.*v*. vs. T39+*P*.*v*.	0.92

Pearson’s correlation coefficients are based on the expression values of all grapevine genes, expressed as fragments per kilobase of transcript per million fragments mapped (FPKM), in control (C), *Trichoderma harzianum* T39-treated (T39), *Plasmopara viticola*-inoculated control (C+*P*.*v*.), and *P*. *viticola*-inoculated T39-treated (T39+*P*.*v.*) plants.

### Grapevine genes differentially expressed during *Trichoderma harzianum* T39-induced resistance

**Figure 2:**
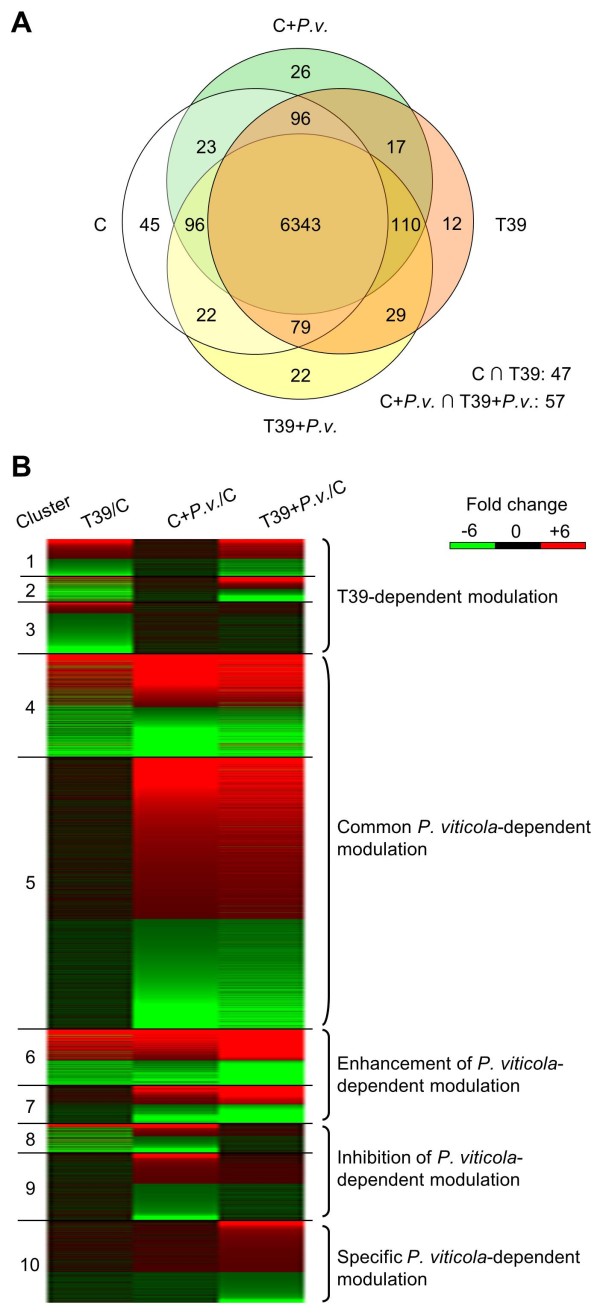
**Grapevine genes differentially expressed during *Trichoderma harzianum *T39-induced resistance to *Plasmopara viticola. ***(**A**) Venn diagram summarising the distribution of 7,024 differentially expressed genes in control (C), T39-treated (T39), *Plasmopara viticola*-inoculated control (C+*P*.*v*.) and *P*. *viticola*-inoculated T39-treated (T39+*P*.*v*.) plants. Differentially expressed genes were identified by the DESeq package [47] with a false discovery rate (FDR) of 5% and a fold-change greater than two in at least one pairwise comparison. (**B**) Grouping of differentially expressed genes into 10 clusters based on the expression profiles. Genes modulated by T39 were distinguished as those with comparable expression levels before and after *P*. *viticola* inoculation in T39-treated plants (cluster 1), genes initially modulated by T39 and further modulated by *P*. *viticola* inoculation in T39-treated plants (cluster 2), and genes affected exclusively by T39 (cluster 3). Genes modulated by *P*. *viticola* with comparable expression levels in control and T39-treated plants were classified as those with (cluster 4) and without (cluster 5) direct modulation by T39. Genes modulated by *P*. *viticola* with greater modulation (> 1.5-fold) in T39-treated than in control plants (ISR-primed genes) where classified as those with (cluster 6) and without (cluster 7) modulation by T39 treatment. Genes modulated by *P*. *viticola* exclusively in control plants were classified as those with (cluster 8) and without (cluster 9) modulation by T39, whereas genes modulated by *P*. *viticola* exclusively in T39-treated plants were grouped in cluster 10 (ISR-responsive specific genes).

DeSeq statistical analysis [47] revealed that 7,024 genes were differentially expressed with respect to the control with a false discovery rate (FDR) of 5% and a minimum fold-change of two in at least one pairwise comparison (Additional file 10). About 90% of these genes were expressed in all treatments (Figure 2a), indicating that T39 and *P*. *viticola* caused a significant modulation (up- or down-regulation greater than 2-fold) of genes normally expressed in control leaves. Relatively few genes were specifically expressed in response to the T39 treatment (12 genes) and after *P*. *viticola* inoculation in control (36 genes) and T39-treated plants (22 genes).

The expression profiles of the differentially expressed genes were determined by cluster analysis, and genes were grouped into 10 clusters based on their expression modulation (Figure 2b and Additional file 10). Among genes modulated by T39, 343 had comparable expression levels before and after *P*. *viticola* inoculation in T39-treated plants (cluster 1), while 233 genes were initially modulated by T39 and then further modulated by *P*. *viticola* inoculation in T39-treated plants (cluster 2). In addition, the expression of 479 genes was affected exclusively by T39 treatment and not by *P*. *viticola* (cluster 3). *P*. *viticola* inoculation resulted in modulation of 3,454 genes with comparable expression levels in control and T39-treated plants at 24 h after inoculation; of these, 948 genes were directly modulated by T39 treatment (cluster 4) and 2,506 genes were not (cluster 5). Interestingly, 868 genes showed reinforced modulation in T39-treated plants compared to control plants after *P*. *viticola* inoculation (ISR-primed genes), indicating enhancement of the grapevine defence reaction to the pathogen. Among these genes, 518 and 350 genes were modulated (cluster 6) or not (cluster 7) by T39 treatment, respectively. A total of 888 genes were modulated by *P*. *viticola* exclusively in control plants; of these, 267 genes were directly modulated by T39 treatment (cluster 8) and 621 genes were not (cluster 9). Conversely, 759 genes were modulated by *P*. *viticola* exclusively in T39-treated plants (cluster 10, ISR-responsive specific genes), and they represent the specific reaction to *P*. *viticola* of T39-treated plants. ISR-responsive specific genes were mainly induced (63%), while genes modulated by *P*. *viticola* exclusively in control plants were mainly repressed (55%).

### Functional annotation of differentially expressed genes

**Figure 3:**
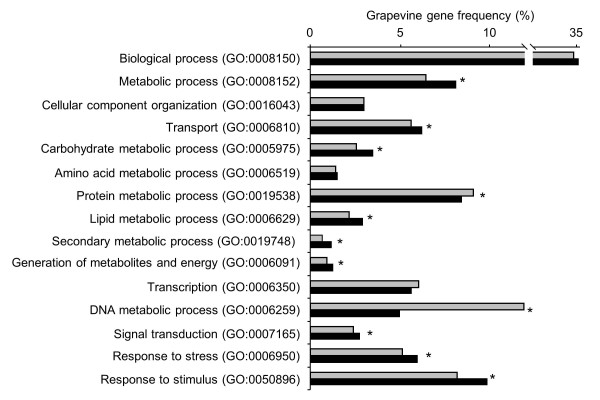
**Annotation of differentially expressed genes. **Distribution of differentially expressed genes (black) and grapevine transcriptome (grey) among the 15 selected Gene Ontology (GO) functional categories (GO identifiers are reported in brackets). Frequencies are calculated as the percentage of the total number of GO biological process terms (8,407 for differentially expressed genes and 39,771 for the grapevine transcriptome) obtained using the ARGOT2 function prediction tool [83,84]. Asterisks indicate GO functional categories differentially represented in the differentially expressed genes compared to the entire grapevine transcriptome according to GOstat statistical analysis (*P* < 0.05) [87].

Differentially expressed genes were automatically annotated and then grouped into 15 functional categories of Gene Ontology (GO) biological processes (Figure 3); genes that could not be associated with any biological process category were assigned to the GO root (biological process). Although the group of genes with unknown function was the largest (35%), 11 categories were represented to different degrees in differentially expressed genes compared to the grapevine transcriptome. DNA (5.0%) and protein (8.4%) metabolic processes were significantly underrepresented among the differentially expressed genes, but response to stress (5.9%), response to stimulus (9.8%), and signal transduction (2.7%) categories were significantly overrepresented. Likewise, transport (6.2%) and metabolism of carbohydrates (3.4%), lipids (2.9%), secondary compounds (1.1%), and energy (1.2%) were significantly overrepresented. The large fraction of differentially expressed genes with unknown function highlighted the identification of novel processes potentially relevant for the induction of plant resistance and for the response of grapevine to *P*. *viticola* inoculation. However, additional studies are required to better characterize the relevance of these genes in the mechanisms of T39-indiuced resistance.

**Figure 4:**
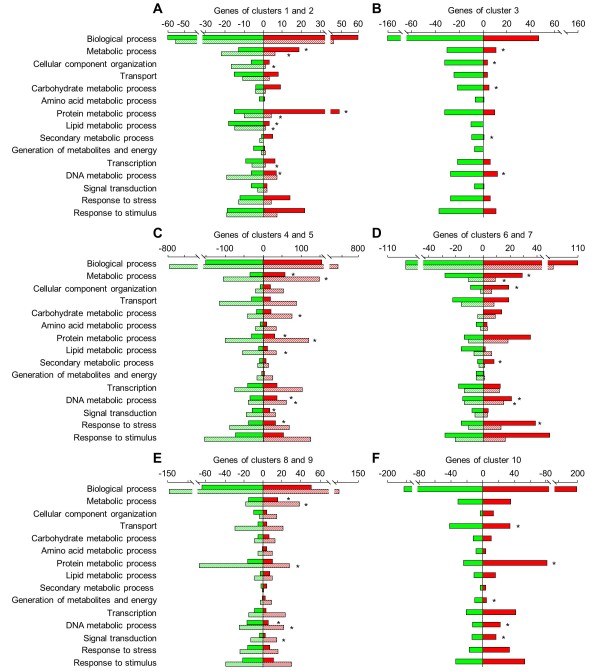
**Functional categories of differentially expressed genes grouped into clusters based on their expression profiles. **Numbers of induced (red) and repressed (green) grapevine genes within each selected Gene Ontology (GO) functional category are reported for each cluster: (**A**) Genes modulated by *Trichoderma harzianum* T39 (T39) with comparable expression levels before and after *Plasmopara viticola* inoculation in T39-treated plants (cluster 1, solid bars) and genes initially modulated by T39 and further modulated by *P*. *viticola* inoculation in T39-treated plants (cluster 2, hatched bars); (**B**) Genes modulated exclusively by T39 treatment (cluster 3); (**C**) Genes similarly modulated by *P*. *viticola* in control and T39-treated plants with (cluster 4, solid bars) or without (cluster 5, hatched bars) direct modulation by T39 treatment; (**D**) Genes modulated by *P*. *viticola* with greater modulation (> 1.5-fold) in T39-treated than in control plants (ISR-primed genes) with (cluster 6, solid bars) or without (cluster 7, hatched bars) direct modulation by T39 treatment; (**E**) Genes modulated by *P*. *viticola* exclusively in control plants with (cluster 8, solid bars) or without (cluster 9, hatched bars) direct modulation by T39; (**F**) Genes modulated by *P*. *viticola* exclusively in T39-treated plants (cluster 10). For each cluster of differentially expressed genes, GO functional categories marked by an asterisk were differentially represented to the entire grapevine transcriptome, according to GOstat statistical analysis (*P* < 0.05) [87].

After genes with unknown function were discounted, a specific distribution of up- and down-regulated functional categories was observed in the various clusters (Figure 4). Genes of cluster 1 involved in carbohydrate, protein, and secondary metabolism were mainly induced by T39 (69, 76 and 83%, respectively), whereas those related to lipid metabolism and transport were mainly repressed (86 and 65%, respectively; Figure 4a). Genes of cluster 2 related to protein metabolism and transcription were mainly repressed by T39 and subsequently induced by *P*. *viticola* inoculation of T39-treated plants. More specifically, T39 treatment directly induced the expression of genes encoding protein kinases and receptor-like protein kinases, MYB transcription factors, hormone-responsive genes, defence-related proteins, and enzymes of secondary metabolism. Annotation of genes modulated exclusively by T39 (cluster 3) revealed down-regulation of processes related to response to stimulus, cellular component organization, and protein metabolism (Figure 4b).

Genes that were similarly modulated by *P*. *viticola* in control and T39-treated plants (clusters 4 and 5) were mainly related to primary metabolic processes and signal transduction (Figure 4c). In particular, genes involved in cellular component organization and carbohydrate metabolism were mainly induced (74 and 65%, respectively). More specifically, *P*. *viticola* induced six cellulose synthase-like genes, six expansin genes, as well as genes encoding enzymes of glucan biosynthesis and hexose metabolism. Conversely, lipid metabolic process, signal transduction, response to stress, and response to stimulus were mainly repressed by *P*. *viticola* (60, 58, 56 and 55%, respectively); this included genes encoding protein kinases, receptor-like protein kinases, disease resistance proteins, and PRs.

The categories of response to stress, secondary metabolism, and DNA metabolism were significantly overrepresented in clusters 6 and 7, and the genes in these categories were mainly up-regulated following *P*. *viticola* inoculation (Figure 4d). In particular, expression of protein kinase, disease resistance protein, chitinase (*Chit*), stilbene synthase (*STS*), resveratrol o-methyltransferase, phenylalanine ammonia-lyase (*PAL*), and transcription factor genes was enhanced after *P*. *viticola* inoculation in T39-treated plants compared with control plants.

Clusters of genes modulated by *P*. *viticola* exclusively in control plants revealed global down-regulation of genes related to protein metabolic process (62 and 71% in clusters 8 and 9, respectively), response to stress (70 and 60%, respectively), and response to stimulus (66 and 57%, respectively; Figure 4e). Interestingly, the same categories repressed by *P*. *viticola* in control plants were globally induced in T39-treated plants (cluster 10; Figure 4f). In particular, genes related to protein metabolic process, response to stress, response to stimulus, and transcription were mainly up-regulated by *P*. *viticola* in T39-treated plants (77, 67, 61, and 67%, respectively). This active reaction to the pathogen in T39-treated plants included expression of protein kinases, transcription factors, auxin and JA/ET signals, phenylpropanoid biosynthesis, and defence-related genes.

### Validation of RNA-Seq analysis

**Figure 5:**
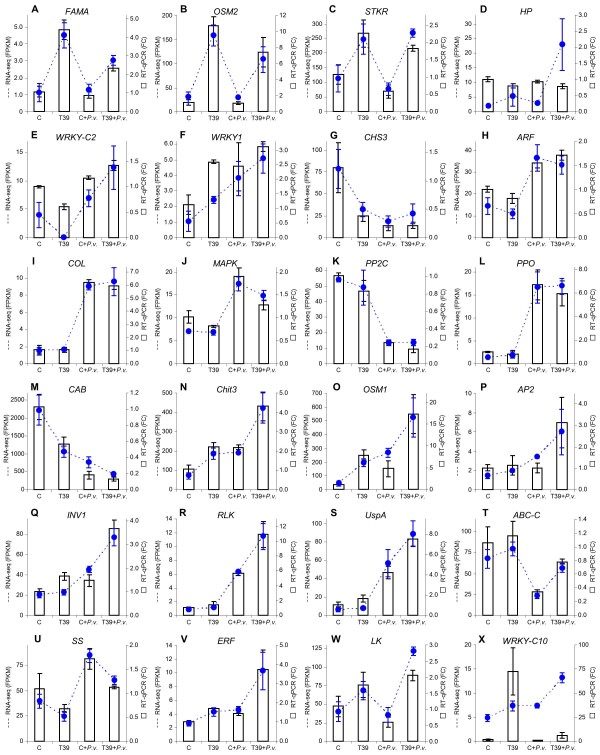
**Comparison of RNA**-**Seq and real**-**time RT**-**PCR analyses. **Expression profiles of (**A**) transcription factor *FAMA*, (**B**) *Trichoderma*-induced osmotin 2 (*OSM2*), (**C**) serine/threonine kinase receptor (*STKR*), (**D**) hypothetical protein (*HP*), (**E**) *WRKY* transcription factor of cluster 2 (*WRKY*-*C2*), (**F**) WRKY transcription factor 1 (*WRKY1*), (**G**) chalcone synthase 3 (*CHS3*), (**H**) auxin response factor (*ARF*), (**I**) constans-like protein (*COL*), (**J**) mitogen-activated protein kinase (*MAPK*), (**K**) protein phosphatase 2C (*PP2C*), (**L**) polyphenol oxidase (*PPO*), (**M**) chlorophyll a-b binding protein (*CAB*), (**N**) acidic endochitinase 3 (*Chit3*), (**O**) *Trichoderma*-induced osmotin 1 (*OSM1*), (**P**) AP2-like ethylene-responsive transcription factor (*AP2*), (**Q**) invertase (*INV1*), (**R**) receptor-like serine/threonine-protein kinase (*RLK*), (**S**) universal stress A-like protein (*UspA*), (**T**) ABC transporter C family member (*ABC*-*C*), (**U**) sucrose synthase (*SS*), (**V**) ethylene-responsive transcription factor (*ERF*), (**W**) LRR receptor-like serine/threonine-protein kinase (*LK*), and (**X**) WRKY transcription factor of cluster 10 (*WRKY*-*C10*). Blue dotted lines represent expression levels (FPKM) as assessed by RNA-Seq analysis and reported as means and standard errors of three biological replicates for each treatment: control (C), *Trichoderma harzianum* T39-treated (T39), *Plasmopara viticola*-inoculated control (C+*P*.*v*.), and *P*. *viticola*-inoculated T39-treated (T39+*P*.*v*.) plants. Histograms represent the relative expression levels (fold-change relative to the expression in control plants) as assessed by real-time RT-PCR and reported as means and standard errors of three biological replicates for each treatment.

To validate the RNA-Seq results, we used real-time RT-PCR to analyse the expression levels of 24 differentially expressed genes (Figure 5). We selected genes with different expression profiles and expression levels, including genes associated with different functional categories (Additional file 11). Although the extent of modulation revealed by real-time RT-PCR and RNA-Seq may differ [6,48,49], the real-time RT-PCR expression profiles in our analysis were in complete agreement with the RNA-Seq data for 19 genes. The expression profiles generated by real-time RT-PCR and RNA-Seq differed for three genes in one treatment and for two genes in all treatments. These differences could be due to differences in sensitivity, particularly in distinguishing members of multigene families. Specificity for RNA-Seq analysis was observed in two grapevine chitinases (Table 4) that are known to be strongly (*Chit1a*) or weakly (*Chit1b*) up-regulated after *P*. *viticola* inoculation [50].

**Table 4:** **Expression levels of grapevine genes known to be modulated during *Trichoderma *spp**.-**induced resistance and in response to *Plasmopara viticola *inoculation**

**Gene name**	**Gene code**^**a**^	**Expression level(FPKM)**^**b**^				**References**^**c**^
		**C**	**T39**	**C+*P.v.***	**T39+*P.v.***	
Chitinase 1a (*Chit1a*)	glimmer.VV78X092424.7_2	13.1	15.1	96.6	66.3	Induced by *P*. *viticola*[50]
Chitinase 1b (*Chit1b*)	glimmer.VV78X202842.8_3	78.4	213.1	167.2	340.5	Not induced by *P*. *viticola*[50]
Pathogenesis-related protein 1 (*PR*-*1*)	fgenesh.VV78X169899.6_1	262.7	379.7	770.3	361.9	Modulated during T39-induced resistance in grapevine [42]
Pathogenesis-related protein 2 (*PR*-*2*)	twinscan.VV78X005385.7_1	214.4	689.8	604.7	1295.0	Modulated during T39-induced resistance in grapevine [42]
Pathogenesis-related protein 4 (*PR*-*4*)	glimmer.VV78X053121.6_1	11.1	99.3	141.3	279.7	Modulated during T39-induced resistance in grapevine [42]
Pathogenesis-related protein 5 (*PR*-*5*)	glimmer.VV79X000230.2_4	65.2	43.2	20.3	11.7	Modulated during T39-induced resistance in grapevine [42]
Pathogenesis-related protein 10 (*PR*-*10*)	sim4.VV78X227342.7_2	1.9	33.9	14.5	34.8	Modulated during T39-induced resistance in grapevine [42]
Lipoxygenase 9 (*LOX*-*9*)	twinscan.VV78X044916.31_1	4.3	6.4	5.0	10.5	Modulated during T39-induced resistance in grapevine [42]
Osmotin 1 (*OSM1*)	glimmer.VV78X132476.3_2	43.6	194.6	268.4	522.1	Induced by *T*. *hamatum* 382 in tomato [29] and induced by *P*. *viticola*[4,6]
Osmotin 2 (*OSM2*)	fgenesh.VV78X075443.24_1	29.7	158.2	29.3	108.6	Induced by *T*. *hamatum* 382 in tomato [29]
Aminocyclopropane carboxylate oxidase (*ACO*)	fgenesh.VV78X260154.9_1	14.8	40.6	22.1	36.0	Induced by *T*. *koningiopsis* Th003 in tomato [54]
Aminocyclopropane carboxylate oxidase (*ACO*)	twinscan.VV78X152558.9_1	23.0	46.6	32.0	50.7	Induced by *T*. *koningiopsis* Th003 in tomato [54]
Trichoderma-induced protein kinase (*TIPK*)	fgenesh.VV78X024841.39_2	60.6	226.8	360.3	374.0	Induced by *T*. *asperelloides* T203 in cucumber [53]
MYB transcription factor 72 (*MYB72*)	fgenesh.VV78X183580.6_2	1.0	2.2	1.9	3.3	Involved in *Trichoderma*-induced resistance [26]
Glucanase (*GLU*)	glimmer.VV78X010792.29_19	4.1	7.5	29.5	17.0	Induced by *P*. *viticola*[5]
Invertase (*INV1*)	fgenesh.VV78X109126.4_1	19.9	22.8	45.1	76.7	Induced by *P*. *viticola*[4,9]
Invertase (*INV2*)	glimmer.VV78X234553.4_1	6.5	5.9	10.2	19.2	Induced by *P*. *viticola*[4,9]
Glutathione S-transferase (*GST*)	glimmer.VV78X070708.13_2	13.5	17.8	21.7	29.9	Induced by *P*. *viticola*[17]
Catalase (*CAT*)	glimmer.VV78X186369.3_2	942.8	1519.4	2840.2	2961.4	Induced by *P*. *viticola*[52]
Enzymatic resistance protein (*ERP*)	glimmer.VV78X101483.19_2	1078.4	909.1	435.8	446.9	Repressed by *P*. *viticola*[5]
Carbonic anhydrase (*CA*)	fgenesh.VV78X143789.26_4	1382.8	435.6	817.8	550.8	Repressed by *P*. *viticola*[5]
Chlorophyll a-b binding protein (*CAB*)	fgenesh.VV78X207753.10_1	2220.1	1054.3	757.7	422.7	Repressed by *P*. *viticola*[4]
Dihydroflavonol 4-reductase (*DFR*)	glimmer.VV78X173356.4_4	101.1	56.1	27.7	29.4	Repressed by *P*. *viticola*[11]
Chalcone synthase (*CHS*)	glimmer.VV78X073177.9_2	55.8	17.3	6.0	9.5	Repressed by *P*. *viticola*[7]
Chalcone synthase (*CHS*)	glimmer.VV78X195981.16_1	54.0	16.2	5.6	5.1	Repressed by *P*. *viticola*[7]
Chalcone synthase (*CHS3*)	fgenesh.VV78X165120.3_1	78.3	32.1	18.2	26.8	Repressed by *P*. *viticola*[7]
Zeaxanthin epoxidase (*ZEP*)	glimmer.VV78X164562.12_3	221.0	152.3	64.3	51.3	Repressed by *P*. *viticola*[9]
B-amylase (*AMY*)	fgenesh.VV78X223906.2_1	3.7	3.2	1.4	1.2	Repressed by *P*. *viticola*[10]
B-amylase (*AMY*)	glimmer.VV78X087147.9_2	57.8	52.7	11.8	12.0	Repressed by *P*. *viticola*[10]
sucrose phosphate synthase (*SPS*)	fgenesh.VV78X130197.5_1	111.8	78.1	32.2	28.8	Repressed by *P*. *viticola*[10]
Phosphoglucan, water dikinase (*PWD*)	fgenesh.VV78X092604.4_1	23.4	17.0	12.1	10.3	Repressed by *P*. *viticola*[10]
Glucose-1-phosphate adenylyltransferase (*GPAT*)	sim4.VV78X153373.11_4	103.6	65.5	39.2	29.3	Repressed by *P*. *viticola*[10]
Threalase	fgenesh.VV78X152339.9_5	1.0	1.3	2.7	2.8	Induced by *P*. *viticola*[10]
α,α-trehalose-phosphate synthase (*TPS*)	glimmer.VV78X200879.5_4	0.3	0.2	0.1	0.1	Repressed by *P*. *viticola*[10]
Phenylalanine ammonia lyase (*PAL*)	glimmer.VV78X178553.4_4	0.0	0.2	0.2	0.3	Induced by *P*. *viticola*[17,50,94]
Stilbene synthase (*STS*)	glimmer.VV78X271796.76_5	0.8	3.0	5.1	7.5	Induced by *P*. *viticola*[11,50,94]
Stilbene synthase (*STS*)	glimmer.VV78X271796.76_1	0.3	2.2	3.3	4.4	Induced by *P*. *viticola*[11,50,94]
Stilbene synthase (*STS*)	glimmer.VV78X257305.4_1	0.5	0.9	2.0	2.8	Induced by *P*. *viticola*[11,50,94]
Stilbene synthase (*STS*)	glimmer.VV78X121741.14_1	0.1	0.7	1.4	2.2	Induced by *P*. *viticola*[11,50,94]
Amine oxidase (*AO*)	twinscan.VV78X119502.7_1	43.8	63.6	167.5	203.0	Induced by *P*. *viticola*[8]
Serine/threonine kinase receptor (*STKR*)	fgenesh.VV78X255341.2_1	112.8	246.8	76.5	268.1	Induced by *P*. *viticola*[8]
Tropinone reductase (*TR*)	fgenesh.VV78X249214.9_1	0.6	1.0	12.8	4.1	Induced by *P*. *viticola*[6]
Galactinol synthase (*GhGolS*)	glimmer.VV78X016811.4_2	0.1	0.0	0.6	0.3	Induced by *P*. *viticola*[6]
Wound-induced protein (*WIN2*)	glimmer.VV78X081378.10_1	7.1	5.2	22.1	6.9	Induced by *P*. *viticola*[6]
β-glucosidase (*BG*)	fgenesh.VV78X148924.9_1	0.1	0.2	2.0	0.7	Induced by *P*. *viticola*[6]
TMV response-related protein (*TMVR*)	fgenesh.VV78X106668.41_2	13.5	7.1	8.6	6.7	Induced by *P*. *viticola*[6]
Polygalacturonase-inhibiting protein (*PGIP*)^d^	glimmer.VV78X089275.7_1	-	-	-	-	Induced by *P*. *viticola*[6]
WRKY transcription factor 21 (*WRKY21*)	fgenesh.VV78X121610.8_2	32.4	19.9	6.5	6.9	Induced by *P*. *viticola*[6]
Ethylene-regulated transcript 2 (*ERT2*)^d^	glimmer.VV78X175653.7_2	-	-	-	-	Induced by *P*. *viticola*[6]
Rapidly elicited Avr9/Cf-9 proteins (*Avr*/*Cf9*)	fgenesh.VV78X009646.12_6	71.5	70.8	17.9	21.7	Modulated by *P*. *viticola*[5]
Rapidly elicited Avr9/Cf-9 proteins (*Avr*/*Cf9*)	fgenesh.VV78X263622.19_2	236.7	117.8	27.1	22.3	Induced by *P*. *viticola*[4]
Harpin-induced protein 1 (Hin1)	glimmer.VV78X225495.6_2	53.7	63.2	12.1	16.0	Induced by *P*. *viticola*[4]
Hypersensitivity-related 203 J (*Hsr203j*)	fgenesh.VV78X137613.4_1	52.3	45.0	7.7	9.1	Induced by *P*. *viticola*[4,12]

^a^ Grapevine genes of the Pinot Noir grapevine genome [77] Release 3 [78].

^b^ Gene expression levels calculated as fragments per kilobase of transcript per million fragments mapped (FPKM) in control (C), *Trichoderma harzianum* T39-treated (T39), *Plasmopara viticola*-inoculated control (C+*P*.*v*.), and *P*. *viticola*-inoculated T39-treated (T39+*P*.*v.*) plants.

^c^ References of previous analyses.

^d^ Genes not included in the set of differentially expressed genes.

We also validated the RNA-Seq method by confirming the expression of previously identified markers of plant response to *Trichoderma* spp. and *P*. *viticola* (Table 4). Our RNA-Seq data confirmed *P*. *viticola*-dependent up-regulation of *PR1*, *PR2*, *PR4*, and *PR10* genes in susceptible grapevines [5,7,11,19,51,52]. Expression profiles of *PR2*, *PR4*, *PR5*, *PR10*, and lipoxygenase 9 (*LOX9*) were consistent with previous real-time RT-PCR data obtained during T39-induced resistance [42]. The RNA-Seq analysis revealed T39-dependent induction of genes that are known to be involved in *Trichoderma*-induced resistance in other systems [26,29,53,54]. Moreover, RNA-Seq analysis confirmed the modulation of genes known to be affected by *P*. *viticola* in grapevines [4-7,9,11,17,52].

## Discussion

RNA-Seq analyses of leaf samples collected before and 24 h after *P*. *viticola* inoculation from T39-treated and control plants resulted in the identification of 7,024 differentially expressed genes. These genes formed 10 clusters of different expression profiles, highlighting the complex transcriptional reprogramming of T39-induced resistance. T39 treatment directly affected the expression of grapevine genes and to a greater extent enhanced grapevine response to *P*. *viticola* inoculation, indicating a dual effect of T39. At the sampling time points selected in this analysis, we showed that a limited number of changes in gene expression were caused by T39 treatment and that more intense transcriptional reprogramming took place after pathogen inoculation. In particular, opposing modulation of genes related to response to stress and protein metabolism was observed in T39-treated and control plants at 24 h after *P*. *viticola* inoculation, indicating inhibition of disease-related processes and induction of active defence in T39-treated grapevines. Based on the expression profiles, genes directly modulated by T39, as well as genes with reinforced or specific modulation in T39-treated plants after pathogen inoculation are strong candidates for activation of plant self-protection and consequent inhibition of disease-related processes and symptoms development.

### Grapevine processes directly affected by *Trichoderma harzianum* T39

Analysis of the expression profiles revealed a set of genes directly modulated by T39 (182 up- and 161 down-regulated) and whose expression was not affected by the subsequent pathogen inoculation (cluster 1). Several genes of cluster 1 were involved in signal transduction processes, indicating that they may be related to the initial events of recognition of the beneficial microorganism and induction of resistance, as recently demonstrated by the proteomic analysis of T39-induced resistance [55]. Enzymes known to mediate microbial recognition and to trigger defence responses in plant species [56] were up-regulated by T39, and these included 66 receptor-like protein kinase genes, three protein kinase genes, and one protein phosphatase gene. In particular, the serine/threonine kinase receptor (*STKR*) was similar to the *Arabidopsis FRK1* induced by bacterial flagellins [57], and a protein kinase was homologous to the *Trichoderma*-induced kinase (*TIPK*) of cucumber [53]. Furthermore, the *STKR* gene (JG391826) is induced by *P*. *viticola* in resistant grapevines [8], suggesting that it plays a crucial role in the activation of specific defence processes against downy mildew.

Our data suggest that resistance induction was also mediated by hormone signalling and transcriptional reprogramming. Genes related to ET metabolism (two 1-aminocyclopropane-1-carboxylate oxidases) were induced by T39 treatment, together with two *MYB* (including *MYB72*), one *FAMA*, and one *NAC* transcription factors. Activation of ET metabolism was in agreement with previous data showing that JA/ET signals are involved in T39-induced resistance [42,58]. In *Arabidopsis*, *MYB72* plays a crucial role in *Trichoderma*-induced resistance [26] and is required in early signalling of rhizobacteria-induced resistance in *Arabidopsis*[59]. The *NAC* gene is involved during oxidative stress [60], and response to oxidative stress after T39 treatment was suggested by modulation of two peroxidases, three glutathione S-transferases (*GSTs*), and one thioredoxin. Moreover, defence-related genes (two *STSs*, a *Chit*, and a cellulose synthase) were also directly induced, indicating that the maximum expression level of these genes was probably reached after T39 treatment and that this was sufficient to contribute to defence against subsequent *P*. *viticola* inoculation. Other defence-related genes were pre-induced by T39 and further induced after *P*. *viticola* inoculation (cluster 2), indicating reinforcement of grapevine defence after pathogen inoculation.

### Enhanced reaction of T39-treated plants to *Plasmopara viticola*

Plant resistance induced by beneficial microorganisms has been associated with faster and/or stronger activation of defence responses after pathogen inoculation [37]. *P*. *vitiocla*-responsive genes with enhanced expression in T39-treated plants were clustered to distinguish between those directly modulated (cluster 6) and those not modulated (cluster 7) by T39 treatment. These expression profiles provide strong support for the view that *Trichoderma* spp. may have a dual effect: it directly stimulates induction of some genes and further reinforces modulation of these and other genes after pathogen inoculation [42]. The dual effect was also reported for defence-gene modulation during resistance induced by sulfated laminarin [17] and for phytoalexin accumulation during resistance induced by *Rheum palmatum* extracts [24].

Enhanced reaction of T39-treated plants included induction of genes related to response to stimulus and response to stress categories, suggesting improvement of signalling pathways and activation of defence reactions in response to pathogen inoculation. In particular, 50 receptor-like kinase genes (35 in cluster 6 and 15 in cluster 7) and six protein kinase genes (one in cluster 6 and five in cluster 7) of signal transduction were primed in T39-treated plants. In agreement with our results, priming was associated with increased expression of mitogen-activated protein kinases (*MPK3* and *MPK6*) in *Arabidopsis* plants [43]. Up-regulation of *MPK* genes was also associated with the reaction of resistant grapevines to *P*. *viticola*[4,6], and post-translational modification may be the additional mechanism for activating *MPK*s in response to pathogens [20]. Moreover, expression of stress-related genes was enhanced in inoculated T39-treated plants, and these genes included *PR2*, *PR4*, *PR5*, *PR10*, *Chit3*, and osmotin (*OSM1*). Similar profiles were observed for *Arabidopsis PR* genes during *T*. *asperelloides* T203 and *Pseudomonas syringae* interactions [30], indicating common mechanisms of *Trichoderma*-induced resistance against biotrophs. Primed profiles were found in resistance-related genes (12 genes in cluster 6 and eight in cluster 7). *Peronospora parasitica* resistance genes (*RPP*) must be expressed at optimal levels to function against downy mildew in *Arabidopsis*[61], and specific profiles of grapevine *RPP* genes suggest fine tuning during T39-induced resistance.

A key role of transcriptional regulation and secondary metabolic processes in T39-induced resistance was also indicated by the priming profiles of four *MYB*, two *WRKY*, and 12 ET-responsive transcription factors (e.g., *AP2*), and of *PAL* and *STSs* genes. Moreover, the category DNA metabolic process was significantly overrepresented in ISR-primed genes. In particular, expression of the histone-lysine N-methyltransferases *SUVR4* and *ATX2* was enhanced after *P*. *viticola* inoculation, reflecting possible involvement of epigenetic modifications [62-64] in T39-induced resistance.

Genes with enhanced expression in T39-treated plants have been previously related to defence against downy mildew in resistant grapevines. This was the case for *PR* genes (*PR2*, *PR4*, *PR10*, and *OSM1*), invertase genes (*INV1* and *INV2*), and genes related to secondary metabolism (*PAL*, *STS*s, *STKR*, copper-containing amine oxidase, polyphenol oxidase, and three resveratrol O-methyltransferases), for which up-regulation was greater in resistant than in susceptible grapevines upon *P*. *viticola* inoculation [4,8,11-14]. The expression profiles of these marker genes suggest that the defence processes usually activated against downy mildew in resistant grapevines are partially stimulated in susceptible plants by T39-induced resistance.

### Disease-related processes inhibited in T39-treated plants

Another important aspect of T39-induced resistance was evidenced by genes modulated by *P*. *viticola* exclusively in control plants (clusters 8 and 9). These genes were mainly down-regulated (57 and 55%, respectively), and they reflect exploitation of cellular resources and/or suppression of defence responses during the compatible interaction. Down-regulation caused by *P*. *viticola* in control plants involved categories of response to stress and primary metabolic processes. Many signalling components (kinase, phosphatase, calmodulin, and calcium signalling), transcription factors (*WRKY* and *MYB*), and disease resistance proteins were repressed, supporting the view that suppression of endogenous signals is required to establish the compatible interaction [5]. Three ABC transporters (e.g., *ABCC*) were also repressed, and suppression of some ABC transporters increases the susceptibility to oomycete pathogens [65]. Specific alteration of carbohydrate metabolism by *P*. *viticola* in control plants was highlighted by modulation of glucosidase, galactosidase, mannosidase, and sucrose synthase (*SS*) genes. Moreover, repressed genes of cluster 9 were classified into the categories of energy metabolism (a phytochrome C, two malic enzymes, and a ribokinase) and defence response (five chitinases, three glucanases, three superoxide dismutases, and a callose synthase), reflecting disease-related process employed by *P*. *viticola* only in control plants. *P*. *viticola* might need to actively suppress plant defences during leaf colonisation through microbial effectors, as demonstrated in other oomycetes [66,67]. Particularly, hemibiotrophic and biotrophic species establish intimate associations with plants [66,67]. To establish infection, these pathogens must suppress the plant defence and manipulate the host metabolism by microbial effectors (virulence factors) that are translocated inside the plant cell or secreted into the extracellular space within plant tissue [66,68]. *P*. *viticola* effector genes have been recently described [69], and modulation of host cell defences through virulence factors in susceptible grapevines has been indicated by histochemical [70] and transcriptomic [4] analyses. In agreement with the phenotypic observations, the specific modulation of grapevine genes in inoculated control but not in inoculated T39-treated plants indicates that T39-induced resistance acts by inhibiting some disease-related processes and probably by interfering with some pathogen-induced processes.

### Specific transcriptional response of T39-treated plants to *Plasmopara viticola*

In contrast to broad down-regulation of genes in control plants (clusters 8 and 9), genes specifically modulated by *P*. *viticola* in T39-treated plants (cluster 10) were mainly induced (63%). These opposing reactions to *P*. *viticola* are particularly evident in genes related to protein metabolism, response to stimulus, and response to stress, which were mainly induced in T39-treated plants and mainly repressed in control plants. Up-regulation of genes associated with these categories has been observed in resistant grapevines [4,13,71], indicating that the defence processes of resistant genotypes could be partially activated in susceptible varieties by T39-induced resistance. In particular, all NBS-encoding resistance (*NBSR*) genes modulated by *P*. *viticola* in control plants (cluster 9) were repressed, whereas those with ISR-responsive specific profiles (cluster 10) or ISR-primed profiles (cluster 7) were mainly up-regulated. Opposing modulation of *NBSR* genes probably reflects suppression of plant defence in control plants and activation of defence responses in T39-treated plants. Interestingly, the *NBSR* genes of clusters 7 and 10 belong mainly to the Va component genome of grapevine [71], indicating subgenome-dependent regulation of gene expression [72] in grapevine.

Defence signals specifically activated in T39-treated plants included those mediated by auxin (two auxin transporters, two auxin-induced proteins, and two indole-3-acetic acid amido synthetases), ET (ACC oxidase and five *ERF* transcription factors), and JA (three lipoxygenases and two fatty acid desaturases). The role of JA/ET signalling pathways in T39-induced resistance has also been demonstrated by phytohormone-affected *Arabidopsis* mutants [58] and by expression analysis of grapevine marker genes [42]. ISR is commonly regulated by JA/ET-dependent signalling pathways, and it is especially active against pathogens deterred by defences that are controlled by JA and ET [33,36]. The auxin response pathway is connected to the SA and JA/ET signalling networks [33], and crosstalk between hormonal pathways [36] enables the fine tuning of defence mechanisms so that the plant can tailor its response to the specific invader [33]. ET exerts its resistance-stimulating activity in concert with JA [33], and JA pathways are involved in the reaction to *P*. *viticola* in resistant grapevines [4,6]. Thus, enhancement of JA/ET signals in T39-treated plants supports the view that increased resistance to downy mildew is mediated by partial activation of extant defence mechanisms normally activated in resistant genotypes. Reaction to the pathogen was also mediated by specific up-regulation of 59 receptor kinases, 10 protein kinases, two *bHLHs* genes, one *MYB* gene, and the *NPR1*.*1* gene.

Our results also suggest that the cell redox balance is altered in T39-treated plants after pathogen inoculation. The reaction of T39-treated plants to pathogen inoculation included the induction four peroxidases and a *GST*. Antioxidant enzymes are often induced in response to pathogens, and alteration of oxidative-stress metabolism has a prominent role in the T39-induced resistance of grapevine to downy mildew [55]. Peroxidases play several important roles in pathogen resistance by contributing to the production of reactive oxygen species, the reinforcement of cell walls, and the production of phytoalexins. Accumulation of stilbene phytoalexin is one of the most important defence processes activated by resistant grapevines in response to *P*. *viticola*[1]; genes of phenylpropanoid biosynthesis (flavanone 3-dioxygenase, laccase, and dihydroflavonol-4-reductase) were specifically induced in T39-treated plants, confirming activation of pathways known in resistant genotypes. However, additional defence mechanisms against *P*. *viticola* are activated in resistant genotypes. In particular, HR-related genes (*Avr*/*Cf9*, *Hin1*, and *Hsr203j*) were not induced and localised HR necrosis was not observed in T39-treated plants.

### Common transcriptional response of control and T39-treated plants to *Plasmopara viticola*

Although specific transcriptional reprogramming of T39-treated plants was observed, 3,454 genes had comparable expression levels in control and T39-treated plants after *P*. *viticola* inoculation (clusters 4 and 5). The pathogen-responsive processes not affected by resistance induction were mainly related to primary metabolism and signal transduction. In particular, expression profiles of genes related to starch metabolism (up-regulation of α-amylase and sugar transporters, down-regulation of β-amylase, glucose-1-phosphate adenylyltransferases, sucrose phosphate synthase, and phosphoglucan water dikinase) indicated the source-to-sink transition of *P*. *viticola*-infected leaves [10]. Likewise, genes related to photosynthesis (two quinone oxidoreductases, a chlorophyllide oxygenase, a protochlorophyllide transporter, a chlorophyllase-2 and chlorophyll a-b binding proteins) and to the Calvin cycle (two rubisco genes and a phosphoglycerate kinase gene) were similarly modulated in inoculated control and T39-treated plants, possibly reflecting the establishment of a compatible interaction. *P*. *viticola* inoculation also resulted in the down-regulation of genes involved in the signal transduction processes (20 receptor-like protein kinase genes, 11 protein kinase genes, and five protein phosphatase genes) and defence response (23 probable disease resistance genes, six unspecified *PR* genes, a *GST* gene and a thaumatin-like gene [73]), indicating a pathogen-dependent suppression of the host reaction mechanisms.

Other markers of *P*. *viticola* infection in susceptible grapevines showed comparable modulation in T39-treated and control plants, such as enzymatic resistance protein [5], zeaxanthin epoxidase [9], catalase [52], isoforms of chalcone synthase [7], and dihydroflavonol 4-reductase [11]. These results suggested that transcriptional changes associated with the compatible interaction [5,7,10,13] were not completely inhibited in T39-treated plants, which is consistent with the observation that downy mildew symptoms were reduced but not completely blocked by T39 treatment.

## Conclusions

The transcriptome analysis reported here represents a major contribution to the characterization of induced resistance mechanisms in a non-model plant. We used the RNA-Seq approach to characterize the transcriptional changes associated with the reduction of downy mildew symptoms in T39-treated grapevines and thereby established a foundation for a more detailed time-course analysis of induced resistance in grapevines. Our data show that T39 directly activates microbial recognition mechanisms in absence of pathogen infection and enhances the expression of defence processes after *P*. *viticola* inoculation. Reduction of downy mildew symptoms is related to inhibition of disease-related processes and activation of defence mechanisms after *P*. *viticola* inoculation of T39-treated plants. T39-induced resistance is associated with enhanced expression of specific genes related to resistance against downy mildew in wild grapevines, indicating that induced resistance can partially mimic defence processes of resistant genotypes. In addition, the genes identified in this work represent an important source of markers of resistance induction and can be used to select beneficial microorganisms with an improved ability to induce plant resistance. These markers can also be used to clarify how environmental conditions affect induced resistance.

## Methods

### Biological materials

Two-year-old plants of the susceptible grapevine (*V*. *vinifera*) cultivar Pinot Noir grafted onto Kober 5BB were individually planted in 2.5 L pots containing a mixture of peat and pumice (3:1). Plants were grown for two months in a greenhouse at 25 ± 1°C with a photoperiod of 16 h light and relative humidity (RH) of 70 ± 10%. *T*. *harzianum* T39 (Trichodex, Makhteshim Ltd., Israel) was applied to grapevine leaves at 8 g L^-1^ in water, corresponding to a conidial suspension of 10^5^ colony-forming units (cfu) mL^-1^. A *P*. *viticola* isolate was collected from an untreated vineyard in the Trentino region (northern Italy) and maintained by subsequent inoculations on *V*. *vinifera* Pinot Noir plants under controlled greenhouse conditions. Plants with oil spot symptoms were incubated overnight in the dark at 99–100% RH. *P*. *viticola* sporangia were collected by washing the abaxial surfaces bearing freshly sporulating lesions with cold distilled water, and the inoculum concentration was adjusted to 10^5^ sporangia mL^-1^ with a haemocytometer.

### Bioassay of induced resistance in grapevine

All leaves of each shoot were treated three times with T39 (at one, two and three days before pathogen inoculation) to induce the greatest phenotypic resistance response [21]. Leaves directly treated with the resistance inducer were analysed to study local effects of T39-induced resistance, and untreated plants were used as control. The suspensions were applied to the abaxial and adaxial leaf surfaces using a compressed air hand sprayer (20–30 mL for each plant).

One day after the last T39 treatment, the abaxial surfaces of all leaves of each plant were inoculated with a *P*. *viticola* suspension (10^5^ sporangia mL^-1^) using a compressed-air hand sprayer (20–30 mL for each plant). All plants were then incubated overnight in the dark at 25 ± 1°C with 99–100% RH and then kept under controlled greenhouse conditions.

Six days after inoculation with the pathogen, all plants were incubated overnight in the dark at 25°C with 99–100% RH. Disease severity was visually assessed as the percentage of abaxial leaf area covered by sporulation [74]. Each treatment was carried out on six replicates (plants). An ANOVA analysis was performed using the Statistica 9 software (StatSoft, Tulsa, OK) followed by Tukey’s test to detect significant differences (*P* < 0.05) in disease severity between treatments.

### Sample collection and RNA isolation

Leaf samples were collected from control and T39-treated plants immediately before and 24 h after *P*. *viticola* inoculation. This time point was chosen because it is associated with leaf colonization by primary hyphae [24,55,75] and with modulation of defence-related genes [4,17,19,42] for the establishment of defence responses in resistant genotypes [4]. Four treatments were analysed by RNA-Seq: control (C), T39-treated (T39), *P*. *viticola*-inoculated control (C+*P*.*v*.), and *P*. *viticola*-inoculated T39-treated (T39+*P*.*v*.) plants. For each treatment, leaf samples from three replicates (plants) were collected at each time point, i.e., three plants per treatment were sampled before inoculation and three plants per treatment were sampled 24 h after inoculation; different plants were sampled at each time point to avoid the effects of wounding stress. Each sample comprised three leaves taken from the same plant, and only leaves of the 4^th^-5^th^ node from the top of the shoot were collected to avoid ontogenic resistance effects [42]. Samples were immediately frozen in liquid N_2_ and stored at −80°C. Total RNA was extracted using the Spectrum Plant total RNA kit (Sigma-Aldrich, St. Louis, MO) and quantified using the Nanodrop 8000 (Thermo Fisher Scientific, Wilmington, DE). The quality of the RNA extracts was checked using the Bioanalyzer 2100 (Agilent Technologies, Santa Clara, CA).

### Library construction and Illumina sequencing

Three biological replicates for each treatment were subjected to RNA-Seq library construction, and each library was sequenced twice in separate lanes (sequencing replicate). In a preliminary experiment, a technical replicate of one sample was run, and the results showed that the library preparation had a high level of technical reproducibility (R^2^ = 0.93, Pearson’s correlation = 0.96). Libraries were prepared using the TruSeq SBS v5 protocol (Illumina, San Diego, CA), and paired-end reads of 100 nucleotides were obtained using an Illumina HiSeq 2000 at Fasteris (Plan-les-Ouates, Switzerland). Briefly, poly (A)-containing mRNA was isolated from 10 μg of total RNA in two rounds of purification using poly-T oligo-attached magnetic beads. Purified mRNA was fragmented using Zn-catalysed hydrolysis and converted into double-stranded cDNA by random priming. Following end repair, indexed adapters were ligated and cDNA fragments of 200 ± 25 bp were purified. Purified cDNA was amplified by PCR and validated by Sanger sequencing, after which mRNA-Seq libraries were multiplexed (six libraries per lane) and sequenced according to the manufacturer’s instructions. The sequences have been deposited at the Sequence Read Archive of the National Center for Biotechnology [76] under BioProject number PRJNA168987.

### Mapping of sequenced reads and assessment of gene expression

For sequence quality control, in-house python scripts were used for quality trimming and sequence filtering: sequencing adapters, k-mers, and bases with a Phred quality score lower than 30 were removed from the read ends, and reads shorter than 50 nucleotides were then discarded. Filtered reads were mapped to the Pinot Noir grapevine genome [77] Release 3 [78] using TopHat 1.2.0 release with default settings [79]; up to 100 hits for each read were allowed to improve gene expression evaluation of members of multigene families [45]. Gene expression values were determined using Cufflinks 1.0.3 release [46], and the FPKM values were calculated for each transcript. Cufflinks default settings were adopted and gene FPKM values were computed by summing the FPKM values of different transcripts of the same gene [46]. The option for fragment bias correction was applied [80], and the RABT assembly option was used to improve the identification of novel genes and transcripts [81]. Sequencing replicates were first analysed separately, and then the read counts of two sequencing replicates were summed and FPKM values recalculated for each biological replicate.

### Differential gene expression analysis

The number of reads falling into each transcript was estimated from the FPKM values according to the formula reported by Zenoni et al. [49], and gene counts were computed by summing counts of different transcripts of the same gene [46]. Differentially expressed genes were identified by the DESeq package [47], with an FDR of the Benjamini-Hochberg multiple tests of 5% (*P* < 0.05) based on read counts and a minimum fold-change of two in at least one pairwise comparison: T39 versus C, C+*P*.*v*. versus C, or T39+*P*.*v*. versus C. Fold-changes were calculated from FPKM expression values with the minimum expression value (10^-4^ FPKM) imposed on non-expressed genes. Genes modulated by pathogen inoculation exclusively in T39-treated plants were classified as ISR-responsive specific genes [38]. Among genes modulated by *P*. *viticola* in control and T39-treated plants, genes that showed an up- or down-regulation greater than 1.5-fold in inoculated T39-treated versus inoculated control plants were selected as ISR-primed genes. This priming cut-off value is based on previous expression data of *Arabidopsis* genes that showed a primed expression profile after pathogen attack [38]. ISR-primed genes were further distinguished as genes directly modulated (cluster 6) or not (cluster 7) by T39 treatment. Principal component analysis (PCA) was performed with Statistica 9 software (StatSoft, Tulsa, OK) on FPKM expression values of all grapevine genes, and the expression profiles of differentially expressed genes were determined by cluster analysis using the T-MeV 4.8.1 software [82].

### Gene annotation

The grapevine genes were automatically annotated using the ARGOT2 function prediction tool [83,84], which was able to annotate grapevine protein sequences by blastp search against the UniProtKB database [85] (downloaded from January 2012) and by association of Gene Ontology biological process terms [86] weighted according to both semantic similarity relations and associated scores [83,84]. Annotation of differentially expressed genes and their distribution over 15 selected functional categories was compared with the entire grapevine transcriptome using GOstat statistical analysis [87]. For manual annotation, grapevine proteins were aligned against the Swiss Prot [88] database (downloaded from February 2012) using blastp, and the best hits (E-value lower than 1E^-5^) were used to select protein descriptions. The corresponding genes of the grapevine PN40024 genotype [89] were identified by blastp search against the 12× release [90] with an imposed E-value lower than 1E^-20^.

### Gene expression analysis by quantitative real-time RT-PCR

Total RNA was treated with DNase I (Invitrogen, Carlsbad, CA), and the first-strand cDNA was synthesized from 1.0 μg of total RNA using Superscript III (Invitrogen) and oligo-dT. Reactions were carried out with Platinum SYBR Green qPCR SuperMix-UDG (Invitrogen) and specific primers (Additional file 11) using the Light Cycler 480 (Roche Diagnostics, Germany). The PCR conditions were: 50°C for 2 min and 95°C for 2 min as initial steps, followed by 50 cycles of 95°C for 15 s and 60°C for 1 min. Each sample was examined in three technical replicates, and dissociation curves were analysed to verify the specificity of each amplification reaction. Light Cycler 480 SV1.5.0 software (Roche) was used to extract Ct values using the second derivative calculation [91], and LinReg software was used to calculate reaction efficiencies [92]. The relative expression of each gene was then calculated according to the Pfaffl equation [93] using control plants as the calibrator. The *actin* gene (glimmer.VV78X114914.6_2) was used as the constitutive gene for normalization [4,52] because its expression was not significantly affected by the treatments (Additional file 12), and comparable results were obtained with *VATP16*[94]. Mean expression and standard error of three biological replicates were calculated for each treatment.

## Competing interests

The authors declare that they have no competing interests.

## Authors’ contributions

MP performed the grapevine treatments, *P*. *viticola* inoculations, RNA extractions, and real-time RT-PCR experiments. MP, MM, PF, and AF analysed the RNA-Seq sequences by mapping, gene expression estimation, and statistical analysis. MP, CM, RV, MD, and IP contributed to data interpretation and manuscript writing. MP, CM, MD, and IP conceived the study, designed the experiment, and coordinated all research activities. All authors read and approved the final manuscript.

## Supplementary Material

Additional file 1 **RNA-Seq sequencing and read mapping of each sequencing replicate. **Numbers of RNA-Seq reads passing the quality check and mapping to the grapevine genome are reported for each sequencing replicate (named A and B) of each biological replicate (numbered from 1 to 3) for control (C), *Trichoderma harzianum* T39-treated (T39), *Plasmopara viticola*-inoculated control (C+*P.v.*), and *P. viticola*-inoculated T39-treated (T39+*P.v.*) plants. Click here for file

Additional file 2 **RNA-Seq sequencing and coverage of the grapevine transcriptome for each biological and sequencing replicate. **Total bases (Mbp) sequenced by RNA-Seq analysis and coverage of the grapevine transcriptome are reported for each sequencing replicate (named A and B) of each biological replicate (numbered from 1 to 3) for control (C), *Trichoderma harzianum* T39-treated (T39), *Plasmopara viticola*-inoculated control (C+*P.v.*), and *P. viticola*-inoculated T39-treated (T39+*P.v.*) plants. Click here for file

Additional file 3 **Distribution of RNA-Seq sequences within the grapevine genome. **(A) Proportions of reads mapping to unique locations (unique reads, yellow), reads mapping to multiple locations with 2–100 matches (multi-reads, red), and reads not mapping or ambiguously mapping to more than 100 locations (unused reads, white) to the grapevine genome. The mean number of sequenced reads and standard errors for three biological replicates are presented for each treatment. Percentages (%) are calculated with respect to the total filtered reads. Unique reads and multi-reads were used for evaluating gene expression. (B) Proportions of reads mapping to grapevine genes (green) and to intergenic regions (red) of the grapevine genome. The mean number of sequenced reads and standard errors for three biological replicates are presented for each treatment. Percentages (%) are calculated with respect to the total mapping reads. Grapevine treatments: control (C), *Trichoderma harzianum* T39-treated (T39), *Plasmopara viticola*-inoculated control (C+*P.v.*), and *P. viticola*-inoculated T39-treated (T39+*P.v*) plants. Click here for file

Additional file 4 **RNA-Seq reads mapping to grapevine genes. **Numbers of RNA-Seq reads mapping to grapevine genes are reported for each sequencing replicate (named A and B) of each biological replicate (numbered from 1 to 3) for control (C), *Trichoderma harzianum* T39-treated (T39), *Plasmopara viticola*-inoculated control (C+*P.v.*), and *P. viticola*-inoculated T39-treated (T39+*P.v.*) plants. Click here for file

Additional file 5 **Expressed grapevine genes and novel genes identified by RNA-Seq analysis. **Numbers of expressed grapevine genes and novel genes with respect to the grapevine annotation are reported for each sequencing replicate (named A and B) of each biological replicate (numbered from 1 to 3) for control (C), *Trichoderma harzianum* T39-treated (T39), *Plasmopara viticola*-inoculated control (C+*P.v.*), and *P. viticola*-inoculated T39-treated (T39+*P.v.*) plants Click here for file

Additional file 6 **Expression levels of grapevine genes. **Gene expression values (FPKM) and standard errors are reported for all (33,514) Pinot Noir grapevine predicated genes [77] Release 3 [78] in control (C), *Trichoderma harzianum* T39-treated (T39), *Plasmopara viticola*-inoculated control (C+*P.v.*), and *P. viticola*-inoculated T39-treated (T39+*P.v.*) plants. Expression values in each biological replicate (numbered from 1 to 3) and each sequencing replicate (named A and B) are also listed. Click here for file

Additional file 7 **Correlations between sequencing replicates of RNA-Seq analysis. **Comparison of the expression levels of all grapevine genes, expressed as fragments per kilobase of transcript per million fragments mapped (FPKM), in the two sequencing replicates (named A and B) of (A) control (C) biological replicate no. 1; (B) C biological replicate no. 2 (B); (C) C biological replicate no. 3; (D) *Trichoderma harzianum* T39-treated (T39) biological replicate no. 1; (E) T39-treated biological replicate no. 2; (F) T39-treated biological replicate no. 3; (G) *Plasmopara viticola*-inoculated control (C+*P.v.*) biological replicate no. 1; (H) C+*P.v.* biological replicate no. 2; (I) C+*P.v.* biological replicate no. 3; (J) *P. viticola*-inoculated T39-treated plants (T39+P.v) biological replicate no. 1; (K) T39+P.v biological replicate no. 2; and (L) T39+P.v biological replicate no. 3. Click here for file

Additional file 8 **Principal component analysis of grapevine treatments. **Principal component analysis (PCA) is based on the expression values (FPKM) of all grapevine genes for each sequencing replicate (named A and B) of each biological replicate (numbered from 1 to 3) for control (C), *Trichoderma harzianum* T39-treated (T39), *Plasmopara viticola*-inoculated control (C+*P.v.*), and *P. viticola*-inoculated T39-treated (T39+*P.v.*) plants. Click here for file

Additional file 9 **Pearson’s correlation coefficients between sequencing and biological replicates of RNA-Seq analysis. **Pearson’s correlation coefficients are based on the gene expression values (FPKM) of all grapevine genes in two sequencing replicates (A and B) of each biological replicate (numbered from 1 to 3) for control (C), *Trichoderma harzianum* T39-treated (T39), *Plasmopara viticola*-inoculated control (C+*P.v.*), and *P. viticola*-inoculated T39-treated (T39+*P.v.*) plants. Click here for file

Additional file 10 **Expression levels, clustering, and annotation results of differentially expressed genes. **Gene expression values (FPKM) and standard errors are reported for differentially expressed (7,024) genes in control (C), *Trichoderma harzianum* T39-treated (T39), *Plasmopara viticola-*inoculated control (C+*P.v.*), and *P. viticola*-inoculated T39-treated (T39+*P.v.*) plants. Differentially expressed genes were identified by the DESeq package with a false discovery rate (FDR) of 5% and a fold-change greater than two in at least one pairwise comparison. Fold-changes, clustering results, and functional annotation are reported for each gene. Click here for file

Additional file 11 **Primer sequences of the grapevine genes analysed by real-time RT-PCR. **Forward and reverse primer sequences of real-time RT-PCR analysis are reported for 24 selected grapevine genes. Grapevine *Actin* (TC81781) [4, 52] and *VATP16* (XM_002269086.1) [94] were used as constitutive genes for normalising the real-time RT-PCR data. Click here for file

Additional file 12 **Expression levels of grapevine genes that are members of the *actin *gene family. **Gene expression values (FPKM) and standard errors are reported for grapevine genes belonging to the *Actin* gene family in control (C), *Trichoderma harzianum* T39-treated (T39), *Plasmopara viticola*-inoculated control (C+*P.v.*), and *P. viticola*-inoculated T39-treated (T39+P.v) plants. Click here for file
