# Supplementary material for: Downy mildew resistance induced by Trichoderma harzianum T39 in susceptible grapevines partially mimics transcriptional changes of resistant genotypes
Source: BMC Genomics. 2012 Nov 22;13:660. doi: 10.1186/1471-2164-13-660 (PMC3551682; doi:10.1186/1471-2164-13-660)
Supplement: Additional file 1 — RNA-Seq sequencing and read mapping of each sequencing replicate. Numbers of RNA-Seq reads passing the quality check and mapping to the grapevine genome are reported for each sequencing replicate (named A and B) of each biological replicate (numbered from 1 to 3) for control (C), Trichoderma harzianum T39-treated (T39), Plasmopara viticola-inoculated control (C+P.v.), and P. viticola-inoculated T39-treated (T39+P.v.) plants. [file 1471-2164-13-660-S1.pdf]

Additional file 1 RNA-Seq sequencing and read mapping of each sequencing replicate

Treatment[a]	Replicate[b]	Sequencing[c]	Total reads	Filtered reads[d]	%	Mapped reads[e]	%	Unique reads[f]	%	Multi-reads[g]	%
C	1	A	8969323	8303055	93	7716851	93	7501989	97	214862	3
	1	B	11511038	11085209	96	10258576	93	9971630	97	286946	3
	2	A	8935961	8278579	93	7786186	94	7552836	97	233350	3
	2	B	11246348	10817280	96	10149194	94	9839646	97	309548	3
	3	A	9741998	9272328	95	8155854	88	7744705	95	411149	5
	3	B	6114294	5573200	91	5171999	93	5004373	97	167626	3
T39	1	A	14212653	13534890	95	12042483	89	11430390	95	612093	5
	1	B	10100086	9673125	96	9061401	94	8777106	97	284295	3
	2	A	16487963	15307482	93	14262870	93	13833343	97	429527	3
	2	B	18827445	17258344	92	16146216	94	15662469	97	483747	3
	3	A	11727855	10864662	93	10039129	92	9708202	97	330927	3
	3	B	13353798	12857723	96	11834839	92	11437356	97	397483	3
C+*P.v.*	1	A	9463465	8780658	93	8134306	93	7866385	97	267921	3
	1	B	12470763	12019827	96	11105833	92	10737272	97	368561	3
	2	A	12033301	11113761	92	10167657	91	9826061	97	341596	3
	2	B	13063851	12570556	96	11470020	91	11070924	97	399096	3
	3	A	8383473	7981653	95	6931957	87	6576463	95	355494	5
	3	B	14087861	13573401	96	12452488	92	12058477	97	394011	3
T39+*P.v.*	1	A	12880128	12186895	95	10136655	83	9584657	95	551998	5
	1	B	10017258	9131790	91	8269752	91	7991903	97	277849	3
	2	A	14725818	13635961	93	12632356	93	12215883	97	416473	3
	2	B	14323785	13074951	91	12168840	93	11765174	97	403666	3
	3	A	9770075	9042028	93	8309902	92	8016077	96	293825	4
	3	B	12833513	12327906	96	11248872	91	10843825	96	405047	4

[a] Grapevine leaves of control (C), *Trichoderma harzianum* T39-treated (T39), *Plasmopara viticola*-inoculated control (C+*P.v.*), and *P. viticola*-inoculated T39-treated (T39+*P.v*) plants.
[b] Biological replicates (plants), numbered from 1 to 3.
[c] Sequencing replicates, named A and B, for each sample.
[d] Reads passing the quality check and the corresponding percentage (%) of total reads.
[e] Reads mapping to the Pinot Noir grapevine genome Release 3 [77], and the corresponding percentage (%) of filtered reads.
[f] Reads mapping to unique locations in the Pinot Noir grapevine genome, and the corresponding percentage (%) of mapped reads.
[g] Reads mapping to more than one location (2-100 matches) in the Pinot Noir grapevine genome, and the corresponding percentage (%) of mapped reads.
