# Supplementary material for: Downy mildew resistance induced by Trichoderma harzianum T39 in susceptible grapevines partially mimics transcriptional changes of resistant genotypes
Source: BMC Genomics. 2012 Nov 22;13:660. doi: 10.1186/1471-2164-13-660 (PMC3551682; doi:10.1186/1471-2164-13-660)
Supplement: Additional file 2 — RNA-Seq sequencing and coverage of the grapevine transcriptome for each biological and sequencing replicate. Total bases (Mbp) sequenced by RNA-Seq analysis and coverage of the grapevine transcriptome are reported for each sequencing replicate (named A and B) of each biological replicate (numbered from 1 to 3) for control (C), Trichoderma harzianum T39-treated (T39), Plasmopara viticola-inoculated control (C+P.v.), and P. viticola-inoculated T39-treated (T39+P.v.) plants. [file 1471-2164-13-660-S2.pdf]

Additional file 2 RNA-Seq sequencing and coverage of the grapevine transcriptome for each biological and sequencing replicate

Treatment[a]	Replicate[b]	Sequencing[c]	Sequenced bases (Mbp)[d]	Read length (bp)[e]	Coverage (fold)[f]
C	1	A	791	95	18
	1	B	1053	95	24
	2	A	787	95	18
	2	B	1025	95	24
	3	A	882	95	20
	3	B	511	92	12
T39	1	A	1290	95	30
	1	B	905	94	21
	2	A	1461	95	34
	2	B	1594	92	37
	3	A	1033	95	24
	3	B	1220	95	28
C+*P.v.*	1	A	838	95	19
	1	B	1143	95	26
	2	A	1054	95	24
	2	B	1190	95	27
	3	A	760	95	17
	3	B	1289	95	30
T39+*P.v.*	1	A	1158	95	27
	1	B	840	92	19
	2	A	1297	95	30
	2	B	1203	92	28
	3	A	860	95	20
	3	B	1168	95	27

[a] Grapevine leaves of control (C), *Trichoderma harzianum* T39-treated (T39), *Plasmopara viticola*-inoculated control (C+*P.v.*), and *P. viticola*-inoculated T39-treated (T39+*P.v*) plants.
[b] Biological replicates (plants), numbered from 1 to 3.
[c] Sequencing replicates, named A and B, for each sample.
[d] Total bases (Mbp) sequenced by RNA-Seq analysis passing quality check.
[e] Mean length (bp) of sequenced reads.
[f] Coverage of the Pinot Noir grapevine transcriptome Release 3 (43.5 Mbp) [77].
