# Supplementary figures and images for: Downy mildew resistance induced by Trichoderma harzianum T39 in susceptible grapevines partially mimics transcriptional changes of resistant genotypes
Source: BMC Genomics. 2012 Nov 22;13:660. doi: 10.1186/1471-2164-13-660 (PMC3551682; doi:10.1186/1471-2164-13-660)

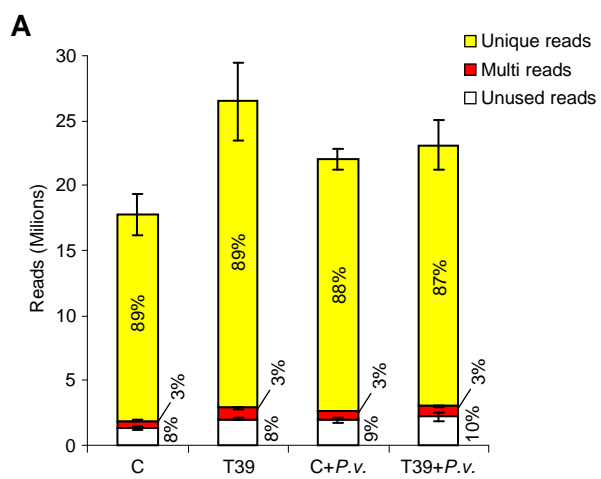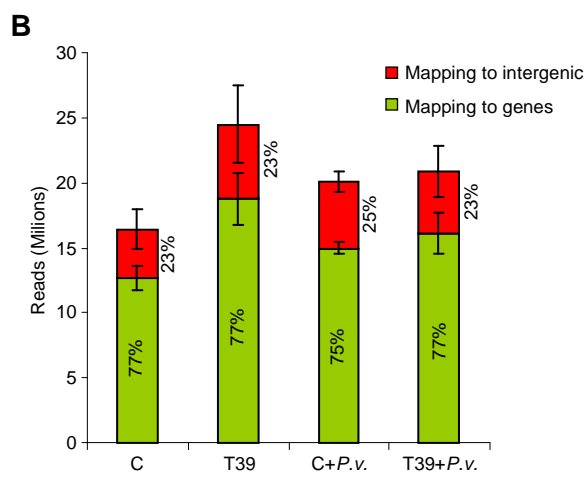

Supplement: Additional file 3 — Distribution of RNA-Seq sequences within the grapevine genome. (A) Proportions of reads mapping to unique locations (unique reads, yellow), reads mapping to multiple locations with 2–100 matches (multi-reads, red), and reads not mapping or ambiguously mapping to more than 100 locations (unused reads, white) to the grapevine genome. The mean number of sequenced reads and standard errors for three biological replicates are presented for each treatment. Percentages (%) are calculated with respect to the total filtered reads. Unique reads and multi-reads were used for evaluating gene expression. (B) Proportions of reads mapping to grapevine genes (green) and to intergenic regions (red) of the grapevine genome. The mean number of sequenced reads and standard errors for three biological replicates are presented for each treatment. Percentages (%) are calculated with respect to the total mapping reads. Grapevine treatments: control (C), Trichoderma harzianum T39-treated (T39), Plasmopara viticola-inoculated control (C+P.v.), and P. viticola-inoculated T39-treated (T39+P.v) plants. [file 1471-2164-13-660-S3.pdf]

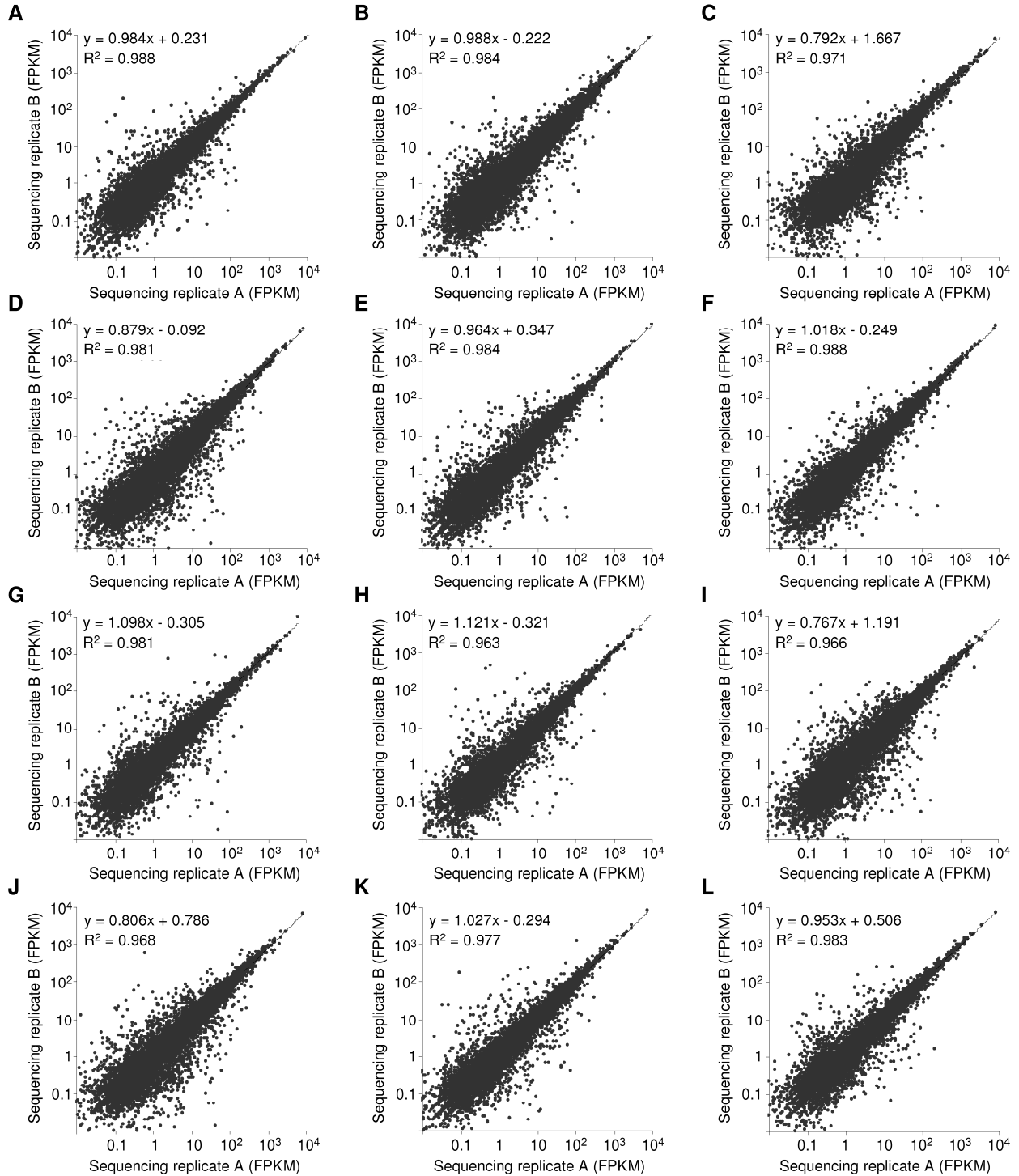

Supplement: Additional file 7 — Correlations between sequencing replicates of RNA-Seq analysis. Comparison of the expression levels of all grapevine genes, expressed as fragments per kilobase of transcript per million fragments mapped (FPKM), in the two sequencing replicates (named A and B) of (A) control (C) biological replicate no. 1; (B) C biological replicate no. 2 (B); (C) C biological replicate no. 3; (D) Trichoderma harzianum T39-treated (T39) biological replicate no. 1; (E) T39-treated biological replicate no. 2; (F) T39-treated biological replicate no. 3; (G) Plasmopara viticola-inoculated control (C+P.v.) biological replicate no. 1; (H) C+P.v. biological replicate no. 2; (I) C+P.v. biological replicate no. 3; (J) P. viticola-inoculated T39-treated plants (T39+P.v) biological replicate no. 1; (K) T39+P.v biological replicate no. 2; and (L) T39+P.v biological replicate no. 3. [file 1471-2164-13-660-S7.pdf]

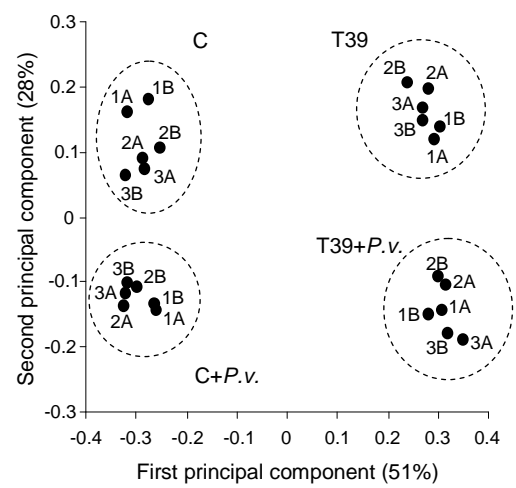

Supplement: Additional file 8 — Principal component analysis of grapevine treatments. Principal component analysis (PCA) is based on the expression values (FPKM) of all grapevine genes for each sequencing replicate (named A and B) of each biological replicate (numbered from 1 to 3) for control (C), Trichoderma harzianum T39-treated (T39), Plasmopara viticola-inoculated control (C+P.v.), and P. viticola-inoculated T39-treated (T39+P.v.) plants. [file 1471-2164-13-660-S8.pdf]
