# Supplementary material for: Downy mildew resistance induced by Trichoderma harzianum T39 in susceptible grapevines partially mimics transcriptional changes of resistant genotypes
Source: BMC Genomics. 2012 Nov 22;13:660. doi: 10.1186/1471-2164-13-660 (PMC3551682; doi:10.1186/1471-2164-13-660)
Supplement: Additional file 4 — RNA-Seq reads mapping to grapevine genes. Numbers of RNA-Seq reads mapping to grapevine genes are reported for each sequencing replicate (named A and B) of each biological replicate (numbered from 1 to 3) for control (C), Trichoderma harzianum T39-treated (T39), Plasmopara viticola-inoculated control (C+P.v.), and P. viticola-inoculated T39-treated (T39+P.v.) plants. [file 1471-2164-13-660-S4.pdf]

Additional file 4 RNA-Seq reads mapping to grapevine genes

Treatment[a]	Replicate[b]	Sequencing[c]	Mapped reads[d]	Reads mapping to genes[e]	%
C	1	A	7716851	5784019	75
	1	B	10258576	7665202	75
	2	A	7786186	5948310	76
	2	B	10149194	7725001	76
	3	A	8155854	6774867	83
	3	B	5171999	3993327	77
T39	1	A	12042483	9909573	82
	1	B	9061401	6791262	75
	2	A	14262870	10669424	75
	2	B	16146216	12115262	75
	3	A	10039129	7801054	78
	3	B	11834839	9126328	77
C+*P.v.*	1	A	8134306	6063846	75
	1	B	11105833	8253396	74
	2	A	10167657	7468646	73
	2	B	11470020	8364086	73
	3	A	6931957	5670434	82
	3	B	12452488	9137073	73
T39+*P.v.*	1	A	10136655	8339148	82
	1	B	8269752	6121404	74
	2	A	12632356	9797665	78
	2	B	12168840	9475386	78
	3	A	8309902	6305104	76
	3	B	11248872	8430951	75
C	1	A+B	17975427	13449221	75
	2	A+B	17935380	13673310	76
	3	A+B	13327853	10768194	81
T39	1	A+B	21103884	16700835	79
	2	A+B	30409086	22784685	75
	3	A+B	21873968	16927382	77
C+*P.v.*	1	A+B	19240139	14317243	74
	2	A+B	21637677	15832732	73
	3	A+B	19384445	14807508	76
T39+*P.v.*	1	A+B	18406407	14460552	79
	2	A+B	24801196	19273052	78
	3	A+B	19558774	14736055	75

[a] Grapevine leaves of control (C), *Trichoderma harzianum* T39-treated (T39), *Plasmopara viticola*-inoculated control (C+*P.v.*), and *P. viticola*-inoculated T39-treated (T39+*P.v*) plants.
[b] Biological replicates (plants), numbered from 1 to 3.
[c] Sequencing replicates, named A and B, and total number as sum of reads obtained by two sequencing replicates (A+B) for each sample.
[d] Reads mapping to the Pinot Noir grapevine genome Release 3 [77].
[e] Reads mapping to Pinot Noir genes Release 3 [77] using Cufflinks [46], and the corresponding percentage (%) of mapped reads.
