# Supplementary material for: Downy mildew resistance induced by Trichoderma harzianum T39 in susceptible grapevines partially mimics transcriptional changes of resistant genotypes
Source: BMC Genomics. 2012 Nov 22;13:660. doi: 10.1186/1471-2164-13-660 (PMC3551682; doi:10.1186/1471-2164-13-660)
Supplement: Additional file 5 — Expressed grapevine genes and novel genes identified by RNA-Seq analysis. Numbers of expressed grapevine genes and novel genes with respect to the grapevine annotation are reported for each sequencing replicate (named A and B) of each biological replicate (numbered from 1 to 3) for control (C), Trichoderma harzianum T39-treated (T39), Plasmopara viticola-inoculated control (C+P.v.), and P. viticola-inoculated T39-treated (T39+P.v.) plants [file 1471-2164-13-660-S5.pdf]

Additional file 5 Expressed grapevine genes and novel genes identified by RNA-Seq analysis

Treatment[a]	Replicate[b]	Sequencing[c]	Expressed genes[d]	%	Novel genes[e]
C	1	A	21499	64	4832
	1	B	21959	66	5709
	2	A	21670	65	4518
	2	B	22085	66	5225
	3	A	22473	67	5259
	3	B	20959	63	3679
T39	1	A	23063	69	7218
	1	B	21651	65	5499
	2	A	22516	67	6939
	2	B	22702	68	7548
	3	A	21785	65	5477
	3	B	22018	66	5890
C+*P.v.*	1	A	21822	65	5502
	1	B	22198	66	6470
	2	A	22454	67	5686
	2	B	22561	67	5979
	3	A	22392	67	5335
	3	B	22427	67	6555
T39+*P.v.*	1	A	22936	68	7333
	1	B	21819	65	5897
	2	A	22873	68	7073
	2	B	22849	68	7082
	3	A	21834	65	5408
	3	B	22242	66	6296

[a] Grapevine leaves of control (C), *Trichoderma harzianum* T39-treated (T39), *Plasmopara viticola*-inoculated control (C+*P.v.*), and *P. viticola*-inoculated T39-treated (T39+*P.v*) plants.
[b] Biological replicates (plants), numbered from 1 to 3.
[c] Sequencing replicates, named A and B, for each sample.
[d] Grapevine genes expressed more than 0 FPKM, and the corresponding percentage (%) of total grapevine genes (33,514) of Pinot Noir Release 3 [77].
[e] Novel genes, with respect to the Pinot Noir Release 3 annotation, identified using Cufflinks [46, 81].
