# Supplementary material for: Downy mildew resistance induced by Trichoderma harzianum T39 in susceptible grapevines partially mimics transcriptional changes of resistant genotypes
Source: BMC Genomics. 2012 Nov 22;13:660. doi: 10.1186/1471-2164-13-660 (PMC3551682; doi:10.1186/1471-2164-13-660)
Supplement: Additional file 9 — Pearson’s correlation coefficients between sequencing and biological replicates of RNA-Seq analysis. Pearson’s correlation coefficients are based on the gene expression values (FPKM) of all grapevine genes in two sequencing replicates (A and B) of each biological replicate (numbered from 1 to 3) for control (C), Trichoderma harzianum T39-treated (T39), Plasmopara viticola-inoculated control (C+P.v.), and P. viticola-inoculated T39-treated (T39+P.v.) plants. [file 1471-2164-13-660-S9.pdf]

Additional file 9 Pearson's correlation coefficients between sequencing and biological replicates of RNA-Seq analysis

Treatment[a]	Replicate[b]	Sequencing replicates[c]	Biological replicates[d]		
			1	2	3
C	1	0.99	1		
	2	0.99	0.99	1	
	3	0.98	0.97	0.97	1
T39	1	0.99	1		
	2	0.99	0.97	1	
	3	0.99	0.98	0.99	1
C+*P.v.*	1	0.98	1		
	2	0.98	0.96	1	
	3	0.98	0.97	0.97	1
T39+*P.v.*	1	0.98	1		
	2	0.99	0.97	1	
	3	0.99	0.97	0.97	1

[a] Grapevine leaves of control (C), *Trichoderma harzianum* T39-treated (T39), *Plasmopara viticola*-inoculated control (C+*P.v.*), and *P. viticola*-inoculated T39-treated (T39+*P.v*) plants.
[b] Biological replicates (plants), numbered from 1 to 3.
[c] Pearson's correlation coefficients based on the gene expression values (FPKM) of all grapevine genes in two sequencing replicates (A and B) of each biological replicate.
[d] Pearson's correlation coefficients based on the gene expression values (FPKM) of all grapevine genes in three biological replicates (1-3) of each biological treatment.
