# Supplementary material for: Downy mildew resistance induced by Trichoderma harzianum T39 in susceptible grapevines partially mimics transcriptional changes of resistant genotypes
Source: BMC Genomics. 2012 Nov 22;13:660. doi: 10.1186/1471-2164-13-660 (PMC3551682; doi:10.1186/1471-2164-13-660)
Supplement: Additional file 12 — Expression levels of grapevine genes that are members of the actin gene family. Gene expression values (FPKM) and standard errors are reported for grapevine genes belonging to the Actin gene family in control (C), Trichoderma harzianum T39-treated (T39), Plasmopara viticola-inoculated control (C+P.v.), and P. viticola-inoculated T39-treated (T39+P.v) plants. [file 1471-2164-13-660-S12.pdf]

Additional_file_12 Expression levels of grapevine genes that are members of the *actin* gene family

Grapevine gene[a]	Expression value (FPKM)[b]				
	**C**	**T39**	**C+*P.v.***	**T39+*P.v.***	**Modulated[d]**
glimmer.VV78X114914.6_2 [c]	50.2 ± 5.1	56.0 ± 2.1	53.6 ± 6.1	54.7 ± 4.6	
glimmer.VV78X204398.3_2	521.9 ± 63.6	518.0 ± 41.4	527.7 ± 26.2	522.5 ± 21.5	
sim4.VV78X148227.11_2	4.6 ± 0.3	3.4 ± 0.4	7.3 ± 0.3	8.2 ± 0.8	*
glimmer.VV78X040868.15_2	21.3 ± 1.5	21.1 ± 1.9	21.3 ± 0.9	24.4 ± 1.0	
fgenesh.VV78X151557.7_1	2.9 ± 0.5	1.2 ± 0.2	2.8 ± 0.8	2.8 ± 0.5	*
fgenesh.VV78X050901.16_2	16.8 ± 3.8	15.8 ± 1.9	21.3 ± 1.4	26.1 ± 2.6	
fgenesh.VV78X079610.25_1	26.1 ± 2.3	35.5 ± 1.8	43.7 ± 1.1	50.5 ± 6.5	

[a] Pinot Noir grapevine genes Release 3 [77] belonging to the *actin* gene family.
[b] Gene expression levels calculated as fragments per kilobase of transcript per million fragments mapped (FPKM) in control (C), *Trichoderma harzianum* T39-treated (T39), *Plasmopara viticola*-inoculated control (C+*P.v.*), and *P. viticola*-inoculated T39-treated (T39+*P.v*) plants.
[c] Grapevine *actin* isoform used as a constitutive gene for normalising the real-time RT-PCR. Expression of *actin* was not affected by the treatments; primer pairs designed on the corresponding Tentative Consensus sequences TC81781 have been previously used in *P. viticola*-inoculated grapevines [4, 52].
[d] Asterisks indicate genes significantly modulated in at least one comparison with a false discovery rate (FDR) of 5% and a fold-change greater than two.
